# Type I interferon signaling in dendritic cells limits direct antigen presentation and CD8^+^ T cell responses against an arthritogenic alphavirus

**DOI:** 10.1128/mbio.02930-24

**Published:** 2024-11-13

**Authors:** Christopher B. Bullock, Leran Wang, Brian C. Ware, Ngan Wagoner, Ray A. Ohara, Tian-Tian Liu, Pritesh Desai, Bjoern Peters, Kenneth M. Murphy, Scott A. Handley, Thomas E. Morrison, Michael S. Diamond

**Affiliations:** 1 Department of Pathology and Immunology, Washington University School of Medicine, St. Louis, Missouri, USA; 2 Department of Medicine, Washington University School of Medicine, St. Louis, Missouri, USA; 3 Department of Immunology and Microbiology, University of Colorado School of Medicine, Aurora, Colorado, USA; 4 Center for Vaccine Innovation, La Jolla Institute for Immunology, La Jolla, California, USA; 5 Department of Medicine, University of California San Diego, La Jolla, California, USA; 6 Department of Molecular Microbiology, Washington University School of Medicine, St. Louis, Missouri, USA; 7 The Andrew M. and Jane M. Bursky Center for Human Immunology, Washington University School of Medicine, St. Louis, Missouri, USA; 8 Center for Vaccines and Immunity to Microbial Pathogens, Washington University School of Medicine, St. Louis, Missouri, USA; Griffith University-Gold Coast Campus, Gold Coast, Queensland, Australia

**Keywords:** alphavirus, viral pathogenesis, interferons, T cells, T-cell immunity

## Abstract

Ross River virus (RRV) and other alphaviruses cause chronic musculoskeletal syndromes that are associated with viral persistence, which suggests deficits in immune clearance mechanisms, including CD8^+^ T-cell responses. Here, we used a recombinant RRV-gp33 that expresses the immunodominant CD8^+^ T-cell epitope of lymphocytic choriomeningitis virus (LCMV) to directly compare responses with a virus, LCMV, that strongly induces antiviral CD8^+^ T cells. After footpad injection, we detected fewer gp33-specific CD8^+^ T cells in the draining lymph node (DLN) after RRV-gp33 than LCMV infection, despite similar viral RNA levels in the foot. However, less RRV RNA was detected in the DLN compared to LCMV, with RRV localizing principally to the subcapsular region and LCMV to the paracortical T-cell zones. Single-cell RNA-sequencing analysis of adoptively transferred gp33-specific transgenic CD8^+^ T cells showed rapid differentiation into effector cells after LCMV but not RRV infection. This defect in RRV-specific CD8^+^ T effector cell maturation was corrected by local blockade of type I interferon (IFN) signaling, which also resulted in increased RRV infection in the DLN. Studies in *Wdfy4*^−/−^, CD11c-Cre *B2m*^fl/fl^, or Xcr1-Cre *Ifnar1*^fl/fl^ mice that respectively lack cross-presenting capacity, MHC-I antigen presentation by dendritic cells (DCs), or type I IFN signaling in the DC1 subset show that RRV-specific CD8^+^ T-cell responses can be improved by enhanced direct antigen presentation by DCs. Overall, our experiments suggest that antiviral type I IFN signaling in DCs limits direct alphavirus infection and antigen presentation, which likely delays CD8^+^ T-cell responses.

**IMPORTANCE:**

Chronic arthritis and musculoskeletal disease are common outcomes of infections caused by arthritogenic alphaviruses, including Ross River virus (RRV), due to incomplete virus clearance. Unlike other viral infections that are efficiently cleared by cytotoxic CD8^+^ T cells, RRV infection is surprisingly unaffected by CD8^+^ T cells as mice lacking or having these cells show similar viral persistence in joint and lymphoid tissues. To elucidate the basis for this deficient response, we measured the RRV-specific CD8^+^ T-cell population size and activation state relative to another virus known to elicit a strong T-cell response. Our findings reveal that RRV induces fewer CD8^+^ T cells due to limited infection of immune cells in the draining lymph node. By increasing RRV susceptibility in antigen-presenting cells, we elicited a robust CD8^+^ T-cell response. These results highlight antigen availability and virus tropism as possible targets for intervention against RRV immune evasion and persistence.

## INTRODUCTION

Arthritogenic alphaviruses are globally important single-stranded, positive sense enveloped RNA viruses in the *Togaviridae* family. These mosquito-transmitted viruses infect millions of people each year, principally in tropical and subtropical regions. The most globally important of these viruses, chikungunya virus (CHIKV), has become endemic in tropical Asia and South America, with large-scale epidemics infecting over a million people in India in 2005 and the Caribbean in 2013 (1). Although less common than CHIKV, the related Ross River virus (RRV) causes musculoskeletal infection in thousands of people annually in Australia (2), with epidemics also occurring in other South Pacific islands (3).

In humans, RRV infection typically causes acute polyarthralgia or polyarthritis (3), with patients also experiencing rash, fever, myalgia, and fatigue. Although constitutional symptoms subside within a few weeks, debilitating musculoskeletal disease can persist for months or even years (4, 5). The chronic phase of RRV infection is thought to be due to persistence of virus, viral RNA, or viral antigen in joint-associated tissues and muscle (6, 7). No specific therapies for arthritogenic alphaviruses exist, and treatment is limited to anti-inflammatory drugs. Although a live-attenuated vaccine (IXCHIQ) was approved in 2023 for CHIKV (8), vaccines for humans have not been authorized for RRV or any other alphavirus.

The lack of complete clearance of an RNA virus without the capacity for a latency transcriptional program has prompted interest in understanding the effectiveness of the immune response to arthritogenic alphavirus infections. Alphaviruses rapidly induce type I interferon (IFN) and IFN-stimulated genes (ISGs) after target or immune cells sense viral RNA using pattern recognition receptors including RIG-I, MDA5, and TLR3 (9). Mice genetically deficient in type I IFN receptor (*Ifnar1*^−/−^) succumb rapidly to infection by RRV or other alphaviruses within 4 days (10, 11), and for CHIKV, bone marrow chimera experiments showed that IFNAR1 expression is required on nonhematopoietic cells for infection control and survival (12). Ly6C^hi^ monocytes and plasmacytoid dendritic cells (pDCs) have been identified as important sources of type I IFNs after sensing RRV infection through MAVS-dependent and -independent signaling pathways (13).

Although innate immune responses limit early stages of alphavirus infection, adaptive immunity is required for clearance. As an example, wild-type (WT) C57BL/6 mice clear CHIKV from the blood by day 7, whereas animals lacking mature B cells (μMT mice) sustain viremia for more than 500 days (14). *Rag1*^−/−^ mice lacking both B and T cells also had prolonged viremia, but levels were approximately 100-fold higher than in μMT mice, suggesting an independent antiviral function of T cells in viral control. However, the role of CD8^+^ T cells in controlling and clearing alphavirus infection, and their relationship to viral persistence, is less well understood. Whereas CD8^+^ T cells are crucial for clearance of other mosquito-transmitted RNA viral infections including flaviviruses (15, 16), in both RRV and CHIKV infections, the viral burden in some musculoskeletal tissues of *Cd8α*^−/−^ mice, which lack CD8^+^ T cells, is similar to that in WT mice, suggesting an unexpectedly limited role of these key immune cells in clearance (17, 18).

Nonetheless, some data suggest that CD8^+^ T cells can have antiviral functions during arthritogenic alphavirus infections. Splenic CD8^+^ T cells from RRV-infected mice at 35 days post-infection (dpi) can lyse RRV-infected fibroblasts *in vitro*, and an RRV-specific CD8^+^ T-cell line eliminated RRV infection in persistently infected macrophages in culture after 15 days (19). *In vivo*, RRV- or CHIKV-specific CD8^+^ T cells can be detected in infected tissues at low levels at 5 dpi with numbers that increase by 10 dpi (17, 18). Even though CHIKV infection appears largely unaffected by CD8^+^ T cells, adoptively transferred CHIKV–peptide pulsed cells can be cleared rapidly in infected mice (18). Rather than a complete inhibition of CD8^+^ T-cell function, there appears to be a defective or delayed response that limits clearance of alphavirus-infected cells.

Here, we benchmarked the CD8^+^ T-cell response against RRV to the Armstrong strain of lymphocytic choriomeningitis virus (LCMV), an RNA arenavirus that promotes an effective CD8^+^ T-cell response (20). When viruses with shared immunodominant (gp33) CD8^+^ T-cell peptide epitopes were used for subcutaneous inoculation, RRV-specific CD8^+^ T-cell responses in the draining lymph node (DLN) were markedly lower than LCMV, despite similar levels of infection in the foot. The distribution of virus in the DLN also was different, especially in the zones associated with T-cell priming. To determine if we could experimentally overcome this deficient RRV-specific CD8^+^ T-cell response, we treated animals in the foot with an anti-IFNAR1 monoclonal antibody (mAb) or used Xcr1-Cre *Ifnar1*^fl/fl^ mice to modulate the infection of antigen-presenting cells in the DLN. These measures resulted in an earlier RRV-specific, but not LCMV-specific, CD8^+^ T-cell response of greater magnitude with enhanced polyfunctional activity. Although infection of anti-IFNAR1-treated wild-type (WT) mice or Xcr1-Cre *Ifnar1*^fl/fl^ mice resulted in higher viral burden in the foot and DLN, the greater antigen load did not fully explain the enhanced RRV-specific CD8^+^ T response since the improvement in effector CD8^+^ T cells was largely retained in anti-IFNAR1-treated *Wdfy4*^−/−^ mice lacking antigen cross-presentation and was not observed in WT mice infected with 1,000-fold higher inoculating doses of RRV. Overall, our results suggest that the type I IFN response after RRV infection limits dendritic cell (DC) infection, direct antigen presentation, and rapid induction of effector antiviral CD8^+^ T cells.

## RESULTS

### CD8^+^ T cells control LCMV but not RRV infection in the foot and spleen

**Fig 1:**
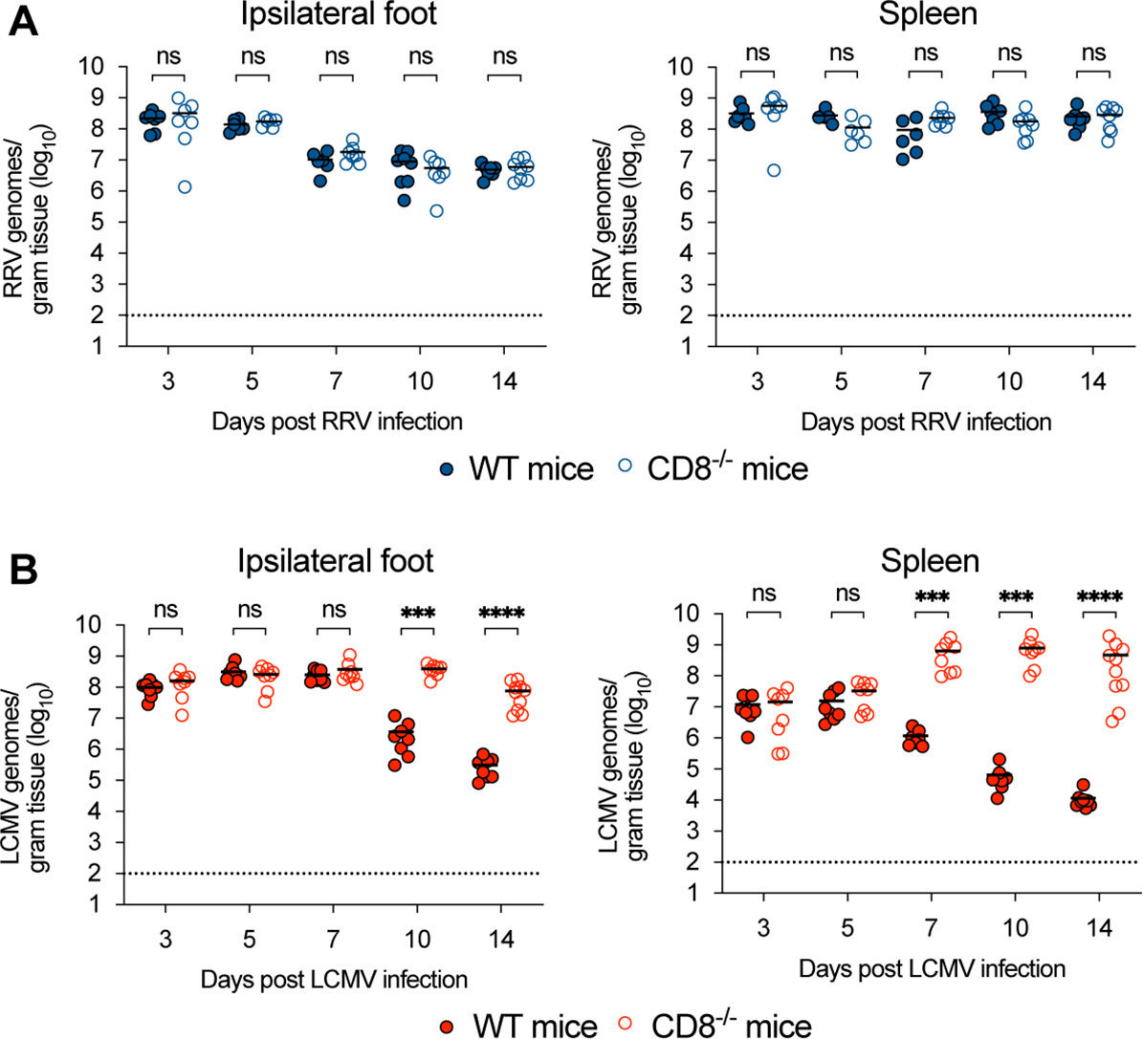
RRV and LCMV infection in wild-type and CD8α^−/−^ mice. Three- to 4-week-old male WT C57BL/6 J or CD8α^−/−^ mice were inoculated in the left footpad with 10^3^ FFU of (**A**) RRV or (**B**) LCMV (*n* = 6–10 mice per group, three experiments). At the indicated day post-infection, tissues were harvested and homogenized. RRV and LCMV viral RNA in the ipsilateral foot (left panels) and spleen (right panels) were titered by qRT-PCR. Statistical analysis: Mann–Whitney test comparing genotypes at each timepoint; ns = not significant; ***P < 0.001; ****P < 0.0001. Bars indicate mean values; dotted lines indicate the limit of detection (LOD).

To begin to understand the relative functional activity of CD8^+^ T-cell responses against alphaviruses, we compared virological outcomes after inoculation of LCMV or RRV at the same infection dose and route in mice having or lacking CD8^+^ T cells. RRV or LCMV (10^3^ focus-forming units [FFU]) was inoculated subcutaneously in the left rear footpad of wild-type (WT) or congenic CD8α^−/−^ C57BL/6 mice. Viral RNA burden was measured in the foot and spleen at 3, 5, 7, 10, and 14 dpi using qRT-PCR. WT and CD8α^−/−^ mice had similar levels of RRV and LCMV RNA at 3 and 5 dpi, respectively (Fig. 1A and B). However, by 7 dpi, a 500-fold difference (*P* < 0.001) in LCMV RNA levels was observed in the spleen, with lower levels in WT than CD8α^−/−^ mice; this difference increased at 10 and 14 dpi. Similarly, in the ipsilateral foot, LCMV RNA levels were lower on days 7, 10, and 14 dpi in WT compared to CD8α^−/−^ mice. In contrast, in RRV-infected mice, viral RNA levels were equivalent in WT and CD8α^−/−^ mice at all timepoints tested (Fig. 1A), indicating the little antiviral effect of CD8^+^ T cells in these tissues.

### Fewer antigen-specific CD8^+^ T cells are generated after RRV than LCMV infection

**Fig 2:**
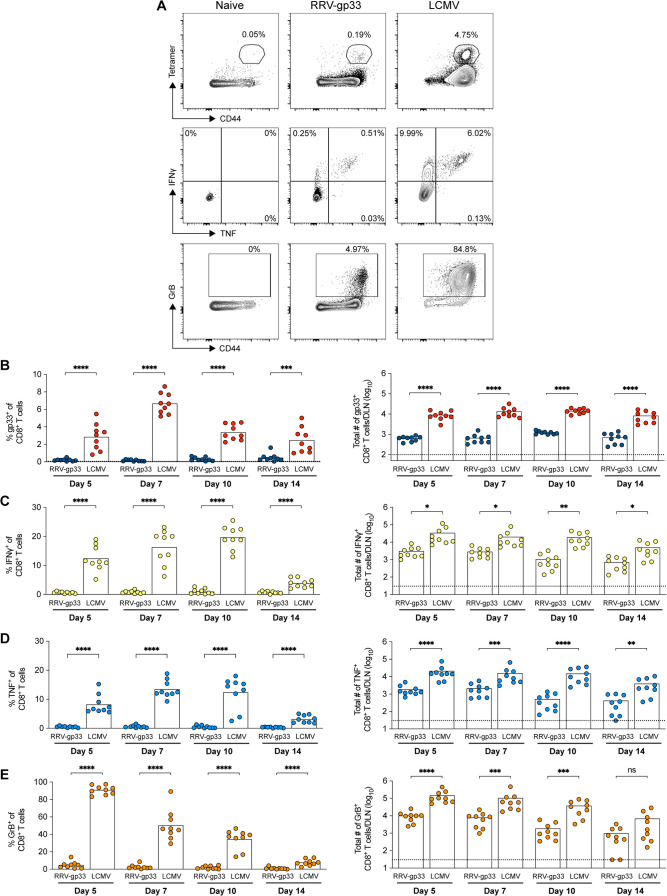
Antigen-specific CD8^+^ T-cell responses in the DLN after RRV and LCMV infection. Three- to 4-week-old male WT C57BL/6 J mice were inoculated in the left footpad with 10^3^ FFU of RRV-gp33 or LCMV (*n* = 9 mice per group, three experiments). At the indicated day post-infection, leukocytes were isolated from the DLN (popliteal), and CD8^+^ T cells were analyzed by flow cytometry. (**A**) Representative flow cytometry contour plots of the gp33 tetramer, IFNγ, TNF, and GrB staining at 5 dpi. (**B–E**) Frequency (left panels) and total numbers (right panels) of CD8^+^ T cells in the DLN. (**B**) Gp33-tetramer positive CD8^+^ T cells. (**C and D**) Cells were restimulated *ex vivo* with gp33 peptide for 4 hours before intracellular staining and flow cytometry for IFNγ^+^ (**C**) or TNF^+^ (**D**) CD8^+^ T cells. (**E**) Granzyme B^+^ CD8^+^ T cells. Statistical analysis: Mann–Whitney test comparing between viruses at each time point; ns = not significant, *P < 0.05, **P < 0.01, ***P < 0.001, and ****P < 0.0001. Column heights indicate mean values; dotted lines indicate the LOD.

To understand why infection by RRV, in contrast to LCMV, was not controlled by CD8^+^ T cells, we profiled the kinetics of the CD8^+^ T-cell response to each virus. To provide the most direct comparison, we utilized a recombinant RRV-gp33 virus that expresses the same immunodominant gp33 peptide epitope as LCMV and replicates to similar levels *in vitro* and *in vivo* as parental RRV (17). We measured CD8^+^ T-cell responses at 5, 7, 10, and 14 dpi against RRV-gp33 and LCMV using a gp33 MHC class I tetramer or after *ex vivo* gp33 peptide restimulation (Fig. 2A; Fig. S1). At 5 dpi, we observed a limited response against RRV-gp33, with approximately 0.3% of total CD8^+^ T cells in the DLN staining with gp33 tetramer (Fig. 2B). In comparison, in LCMV-infected animals, 3% of CD8^+^ T cells in the DLN were stained with the gp33 tetramer. At 7 days after LCMV infection, the proportion of gp33-specific CD8^+^ T cells increased in the DLN before contracting by 10 and 14 dpi, although the overall number of gp33-specific CD8^+^ T cells in the DLN remained relatively constant. LCMV infection induced substantial fractions and numbers of CD8^+^ T cells that expressed IFNγ (Fig. 2C) and TNF (Fig. 2D) after gp33 peptide restimulation, with more antigen-specific cells enumerated than with the less sensitive tetramer binding assay. Granzyme B (GrB) expression was detected without peptide restimulation in >80% of all DLN CD8^+^ T cells at 5 days after LCMV infection (Fig. 2E). In contrast to the robust response seen with LCMV, RRV-gp33 infection induced tenfold lower levels of IFNγ and TNF producing CD8^+^ T cells after peptide restimulation, and GrB expression was apparent in only 5% of DLN CD8^+^ T cells at 5 dpi. Overall, despite similar levels of RRV and LCMV infection in the foot, we detected fewer antigen-specific, cytokine-producing, and GrB-expressing CD8^+^ T-cell responses within the first week of RRV infection.

### CD8^+^ T cells after RRV infection are less activated than after LCMV infection

**Fig 3:**
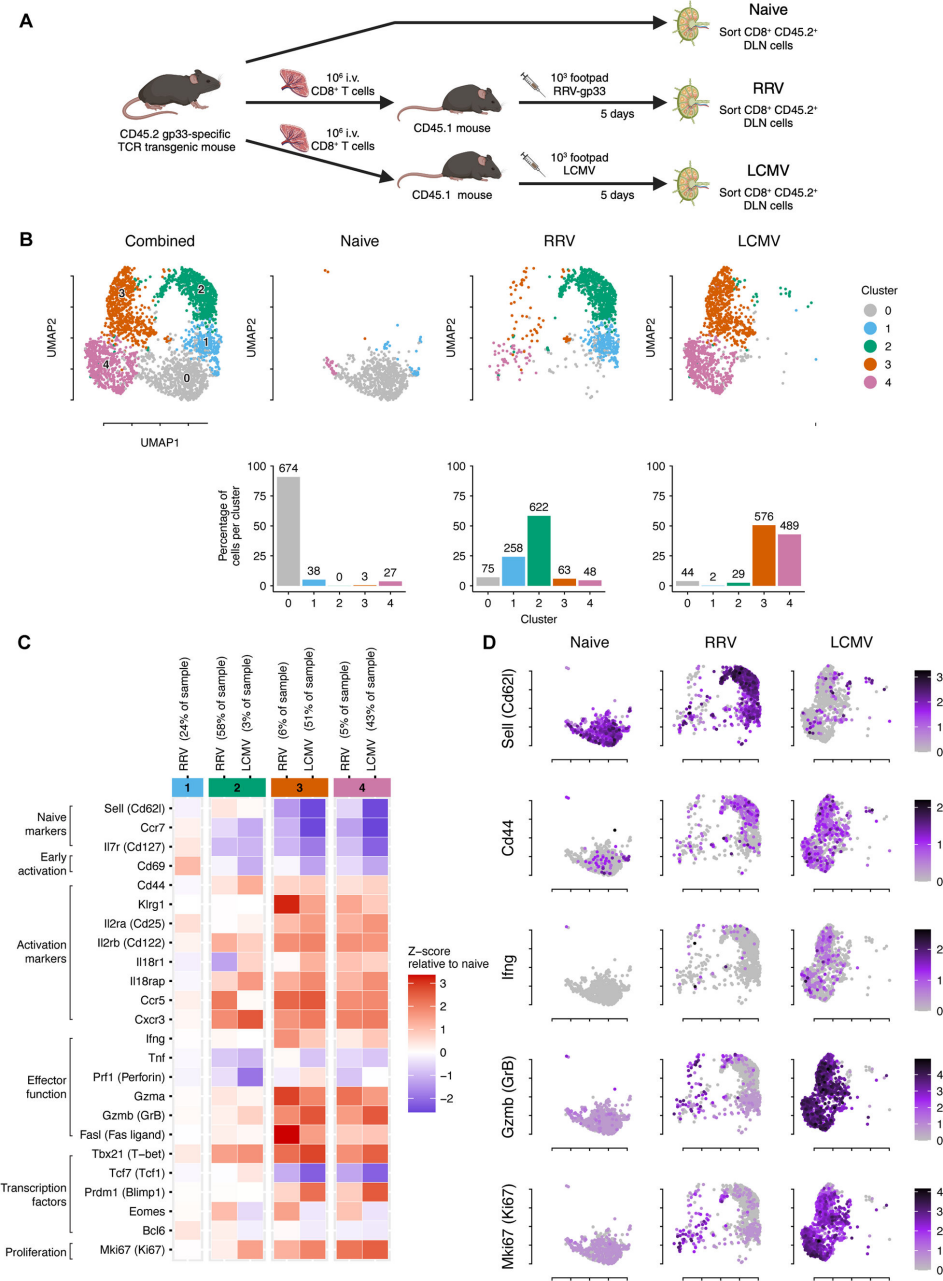
Transcriptomic analysis of transferred gp33-specific CD8^+^ T cells after RRV and LCMV infection. Three- to 4-week-old male WT C57BL/6 J mice were adoptively transferred to 10^6^ gp33-specific P14-transgenic CD8^+^ T cells and then inoculated in the left footpad with 10^3^ of RRV-gp33 or LCMV (*n* = 7 mice per group, one experiment). Five days later, leukocytes were isolated from DLNs and pooled for each respective group. Gp33-specific CD8^+^ T cells were enriched via sorting, and single-cell RNA sequencing was done using the 10X Genomics platform. (**A**) Experimental design. (**B**) Clustering of isolated gp33-specific CD8^+^ T cells and counts per cluster. (**C**) Change in the expression of CD8^+^ T-cell activation genes, per cluster/sample, relative to the mean expression in cluster 0 of the naive sample. (**D**) Expression of selected CD8^+^ T-cell activation genes.

Because of the quantitative and functional defects associated with gp33-specific CD8^+^ T cells after RRV infection, we hypothesized there might be defects in the activation process. To address this question, we adoptively transferred 10^6^ CD8^+^ T cells from donor P14-transgenic mice (which express a T-cell receptor specific for the gp33 peptide) (21) into recipient WT mice, followed by infection with RRV-gp33 or LCMV. Five days later, gp33-specific CD8^+^ T cells were sorted from the DLN of naive transgenic or infected recipient WT mice and subjected to single-cell and 5' T-cell receptor RNA sequencing to confirm gp33-specificity (Fig. 3A). We combined the samples, clustered the cell groups, and plotted them in uniform manifold approximation and projection (UMAP) space. Differential gene expression analysis (Fig. S2) revealed five clusters that we assigned as 0, 1, 2, 3, and 4 (Fig. 3B). Over 90% of the naive P14 CD8^+^ T cells grouped in cluster 0. In RRV- and LCMV-infected mice, only 7% and 5% of CD8^+^ T cells, respectively, were in cluster 0, suggesting that most cells receive some activating signaling in the respective LN environments. After RRV infection, 83% of the CD8^+^ T cells grouped into clusters 1 and 2, which was largely unique to RRV as only 3% of cells were in these clusters after LCMV infection. The majority (93%) of P14 CD8^+^ T cells after LCMV infection grouped into clusters 3 and 4, with a minority (~10%) of cells clustering in these groups after RRV infection. Based on UMAP analysis, the transcriptional fate of transferred P14 CD8^+^ T cells at 5 dpi appears different after RRV and LCMV infections.

We next compared the expression of markers of T-cell activation in the P14 T cells isolated after RRV and LCMV infections to those in cluster 0 from naive mice (Fig. 3C). CD8^+^ T cells in clusters 1 and 2, seen primarily after RRV infection, retained expression of genes associated with naive cells, including migratory capacity (*Ccr7 and Sell* [CD62l]) and survival (*Il7r* [CD127]). Cells in cluster 2 appear to transition toward activation with upregulation of *Cd44*, *Ccr5*, *Cxcr3*, and *Tbx21* (T-bet) transcripts. In clusters 3 and 4, which feature P14 CD8^+^ T cells predominantly after LCMV infection, additional CD8^+^ T-cell activation markers, including *Il2ra* (CD25) and *Klrg1*, were present, suggesting a more terminal effector phenotype (22, 23). Cells in clusters 3 and 4 also showed upregulated expression of effector molecules, including *Ifng*, *Grzmb*, and *Fasl* and the proliferation marker *Mki67* (Ki67). Cells in clusters 1 and 2 did not appear to be intrinsically inhibited as they expressed similar levels of costimulatory (e.g., *Icos* and *Cd28*) (Fig. S3A) and inhibitory receptors (e.g., *Lag3*, *Ctla4,* and *Pd-1*) (Fig. S3B) as cells in clusters 3 and 4. Overall, gp33-specific CD8^+^ T cells in the DLN of RRV-infected mice show evidence of some activation by 5 dpi, but their trajectory toward maturation into effector cells appears deficient or delayed compared to after LCMV infection.

### Weak CD8^+^ T-cell response correlates with low levels and distinct spatial location of RRV infection in the DLN

**Fig 4:**
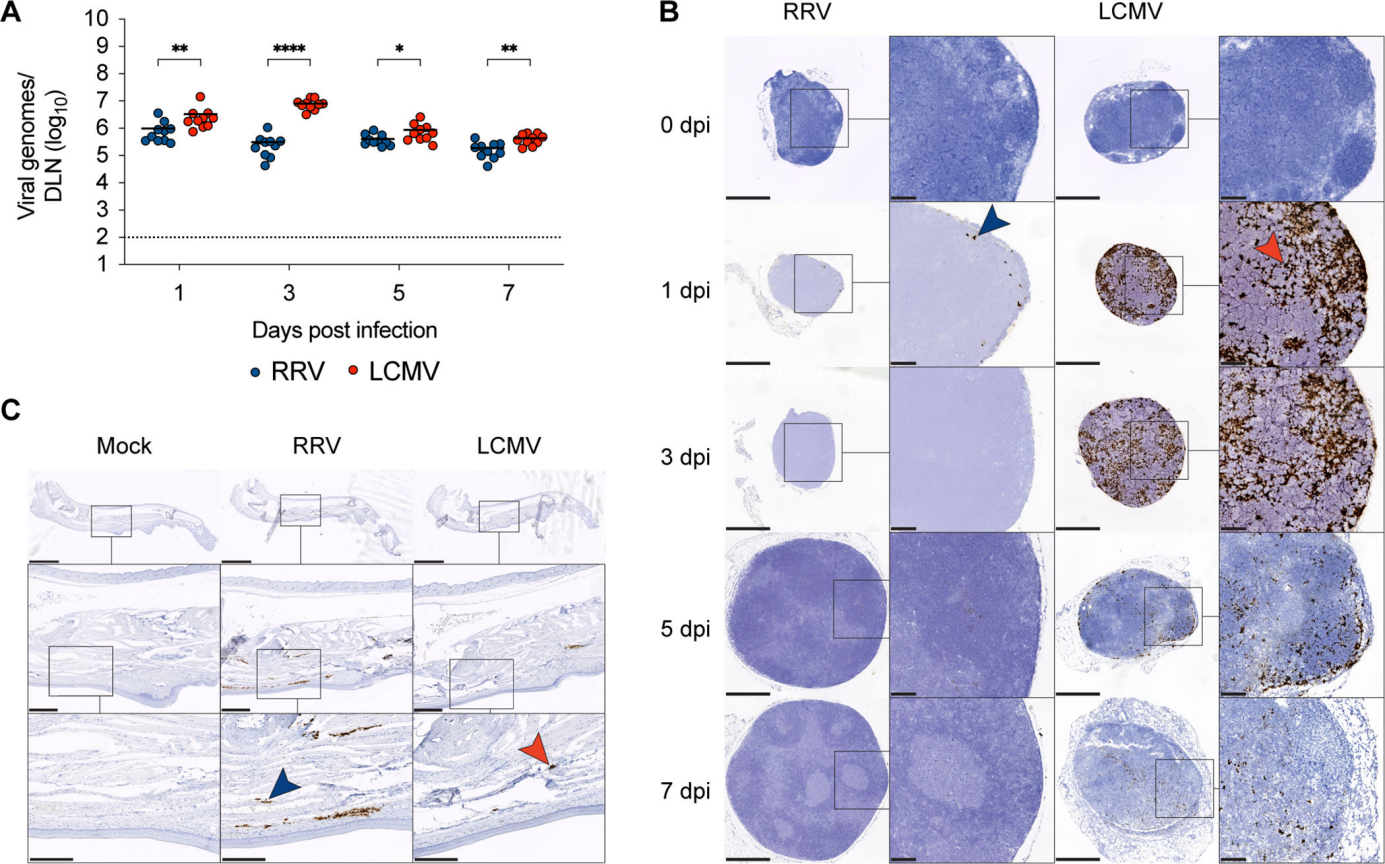
RRV and LCMV RNA infection and localization in the DLN. Three- to 4-week-old male WT C57BL/6 J mice were inoculated in the left footpad with 10^3^ FFU of RRV or LCMV. At the indicated day post-infection, DLNs were harvested. (**A**) RRV and LCMV RNA levels (*n* = 10 mice per group, three experiments). (**B**) Representative images of RRV and LCMV viral RNA localization at 0, 1, 3, 5, or 7 dpi as stained by *in situ* hybridization (scale bars: 500 µm) and high-magnification insets (scale bars: 100 µm) (*n* = 3 mice per group, three experiments). Blue arrow, RRV RNA; red arrow, LCMV RNA. (**C**) Representative images of RRV and LCMV viral RNA localization in the ipsilateral foot at 5 dpi, as stained by *in situ* hybridization (scale bars: 2.5 mm), medium-magnification insets (scale bars: 500 µm), and high-magnification insets (scale bars: 250 µm) (*n* = 3 mice per group, two experiments). Blue arrow, RRV RNA; red arrow, LCMV RNA. Statistical analysis: Mann–Whitney test comparing between viruses at each time point; *P < 0.05, **P < 0.01, and ***P < 0.0001. Bars indicate mean values; dotted lines indicate the LOD.

To begin to understand the basis for the differences in CD8^+^ T-cell responses after LCMV and RRV infection, we compared the levels of virus in the DLN. We hypothesized that less viral infection in the DLN of RRV-infected mice might delay CD8^+^ T-cell priming and expansion. Indeed, levels of RRV RNA in the DLN were 5 to 25-fold lower (*P* < 0.05) than that observed for LCMV over the first week of infection (Fig. 4A). As a recent study had shown that the majority of alphavirus RNA in the DLN is present in the lymphatic fluid rather than the parenchyma (24), we performed *in situ* hybridization at different days after RRV and LCMV infection to determine the spatial location of viral RNA in DLN cells. At 1 dpi, limited amounts of RRV RNA were present, principally in the subcapsular region of the DLN (Fig. 4B). At 3 dpi and later, little-to-no RRV RNA signal was detected, and when present, it appeared to localize to germinal centers. At 5 dpi, while RRV RNA was no longer visibly detected in the DLN by *in situ* hybridization, viral RNA signal was still apparent in the foot (Fig. 4C). These results contrasted with LCMV infection where on 1 dpi, the LCMV RNA signal was apparent throughout the DLN, and at 3, 5, and 7 dpi, LCMV RNA was still visible in the paracortical areas where antigen-presenting cells activate CD8^+^ T cells.

### RRV-specific CD8 ^+^ T-cell priming is increased with the blockade of type I IFN signaling

**Fig 5:**
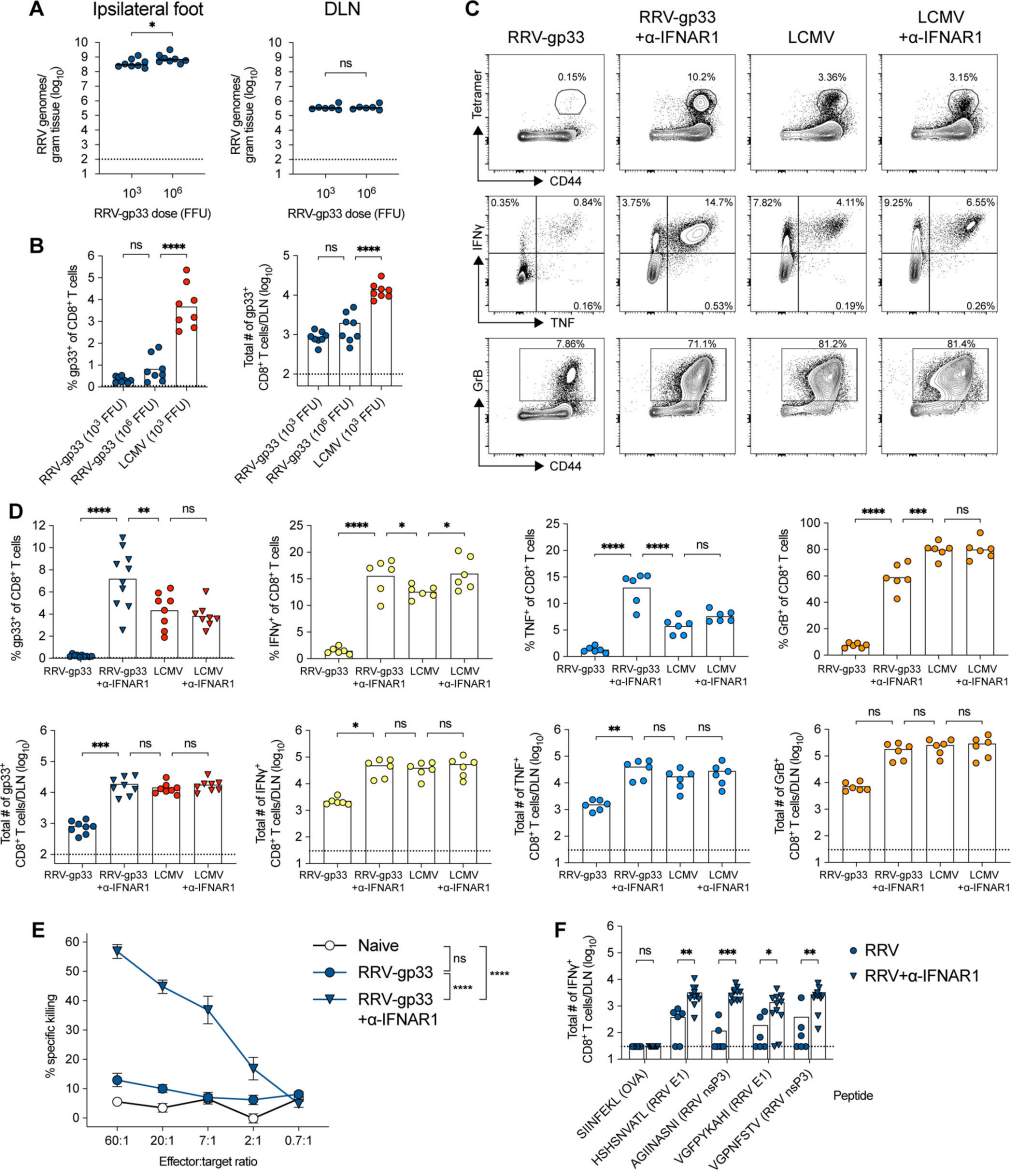
Local treatment with anti-IFNAR1 blocking antibody augments RRV-specific CD8^+^ T-cell responses in the DLN. Three- to 4-week-old male WT C57BL/6 J mice were inoculated in the left footpad and sacrificed at 5 dpi. (**A and B**) Inoculation with 10^3^ FFU of RRV-gp33, 10^6^ FFU of RRV-gp33, or 10^3^ FFU LCMV (*n* = 6–8 mice per group, three experiments). (**A**) RRV RNA in the ipsilateral foot (left panel) and DLN (right panel) was titered by qRT-PCR. (**B**) Gp33-tetramer-positive CD8^+^ T cells in the DLN: frequency (left panels) and total numbers (right panels). (**C and D**) Inoculation with 10^3^ FFU of RRV-gp33 or LCMV, with or without anti-IFNAR1 blocking mAb (α-IFNAR1, 100 µg; 6 mg/kg) treatment administered in the footpad (*n* = 6–10 mice per group, three experiments). (**C**) Representative flow cytometry contour plots of the gp33 tetramer, IFNγ, TNF, and GrB staining in the DLN. (**D**) Summary data in of gp33-tetramer^+^, IFNγ^+,^ and TNF^+^ staining after *ex vivo* peptide restimulation and GrB^+^ CD8^+^ T cells in the DLN: frequency (top panels) and total numbers (bottom panels). (**E**) Inoculation with 10^3^ FFU of RRV-gp33, with or without anti-IFNAR1 mAb treatment (*n* = 7 mice per group, three experiments). CD8^+^ T cells isolated from the DLN at 5 dpi were incubated with labeled naive splenocytes pulsed with 5 µg/mL of gp33 or OVA peptide. After 4 hours, cells were stained with 7-AAD to determine the percent killing of gp33-peptide-pulsed cells by gp33-specific CD8^+^ T cells. (**F**) Inoculation with 10^3^ FFU of RRV, with or without anti-IFNAR1 mAb treatment (*n* = 6–11 mice per group, three experiments). Total numbers of IFNγ^+^ CD8^+^ T cells in the DLN after *ex vivo* peptide restimulation. Statistical analysis: (**A**) Mann–Whitney test; (**B**) one-way ANOVA with Holm–Sidak’s post-test, comparisons between 10^3^ FFU RRV-gp33 and 10^6^ FFU RRV-gp33 and between 10^6^ FFU RRV-gp33 and 10^3^ LCMV; (**D**) one-way ANOVA with Holm–Sidak’s post-test, comparisons between RRV-gp33 and RRV-gp33 + anti-IFNAR1, RRV-gp33 + anti-IFNAR1 and LCMV, and LCMV and LCMV + anti-IFNAR1; (**E**) two-way ANOVA with Holm–Sidak’s post-test, comparisons between naive and RRV-gp33, naive and RRV-gp33 + anti-IFNAR1, and RRV-gp33 and RRV-gp33 + anti-IFNAR1; (**F**) Mann–Whitney test with Holm–Sidak’s post-test comparing with and without anti-IFNAR1 treatment for each peptide; ns = not significant, *P < 0.05, **P < 0.01, ***P < 0.001, and ****P < 0.0001. Bars and column heights indicate mean values; dotted lines indicate the LOD.

Because of the difference in the location of viral RNA in the DLN between RRV and LCMV, we hypothesized that higher amounts of LCMV infection or viral proteins in antigen-presenting cells in the DLN might explain the greater antigen-specific CD8^+^ T-cell response. Indeed, published studies show that LCMV can infect and replicate in conventional DCs in lymphoid tissues (25). In an attempt to experimentally enhance RRV infection in the DLN and antigen presentation, we first inoculated mice with a 1,000-fold higher dose of RRV (10^6^ FFU). However, at 5 dpi, infection was only twofold higher in the foot (*P* < 0.05) and unchanged in the DLN (Fig. 5A). Accordingly, this higher dose of RRV did not statistically increase the number of RRV-specific CD8^+^ T cells (Fig. 5B).

As alphaviruses are sensitive to type I IFN restriction (26), we hypothesized that the blockade of IFN signaling with an anti-IFNAR1 monoclonal antibody (mAb), MAR1-5A3 (27), might increase the infection in the foot and DLN and accelerate the antigen-specific CD8^+^ T-cell response. We administered the blocking mAb locally in the footpad at the time of infection using a nonlethal dose (6 mg/kg) to limit the systemic effects of anti-IFNAR1 treatment, including RRV dissemination to the brain and fatal infection. This blockade of type I IFN signaling resulted in greater CD8^+^ T-cell responses in the DLN at day 5 after RRV-gp33 infection (Fig. 5C), such that the number of gp33-specific, IFNγ^+^, TNF^+^, and GrB^+^ CD8^+^ T cells after RRV infection now matched those of LCMV infection (Fig. 5D). In comparison, the anti-IFNAR1 blockade had little-to-no effect on the magnitude or quality of the CD8^+^ T-cell response after LCMV infection. The improved antigen-specific response in RRV-gp33-infected mice that received anti-IFNAR1 also led to greater lysis of gp33-peptide-pulsed cells by bulk CD8^+^ T cells compared to those isolated from mice infected with RRV-gp33 but not treated with anti-IFNAR1 (Fig. 5E).

To confirm that the increased CD8^+^ T-cell response against RRV after anti-IFNAR1 treatment is not restricted to an immunodominant exogenous peptide epitope, we generated a library of 19 computationally predicted H-2^b^ binding peptides corresponding to different sites in the RRV proteome (see Materials and Methods). After *ex vivo* restimulation of cells from the DLN, we identified four peptides (E1-HSHSNVATL; E1-VGFPYKAHI; nsp3-AGIINASNI; and nsp3-VGPNFSTV) that stimulated CD8^+^ T-cell responses *ex vivo* (Fig. S4), which suggests that the CD8^+^ T-cell response to RRV is not hindered by a lack of immunogenic epitopes. After *ex vivo* restimulation with each of the four RRV peptides, approximately tenfold higher numbers of IFNγ^+^ CD8^+^ T cells were obtained from DLN cells harvested at 5 dpi after local anti-IFNAR1 mAb treatment (Fig. 5F).

**Fig 6:**
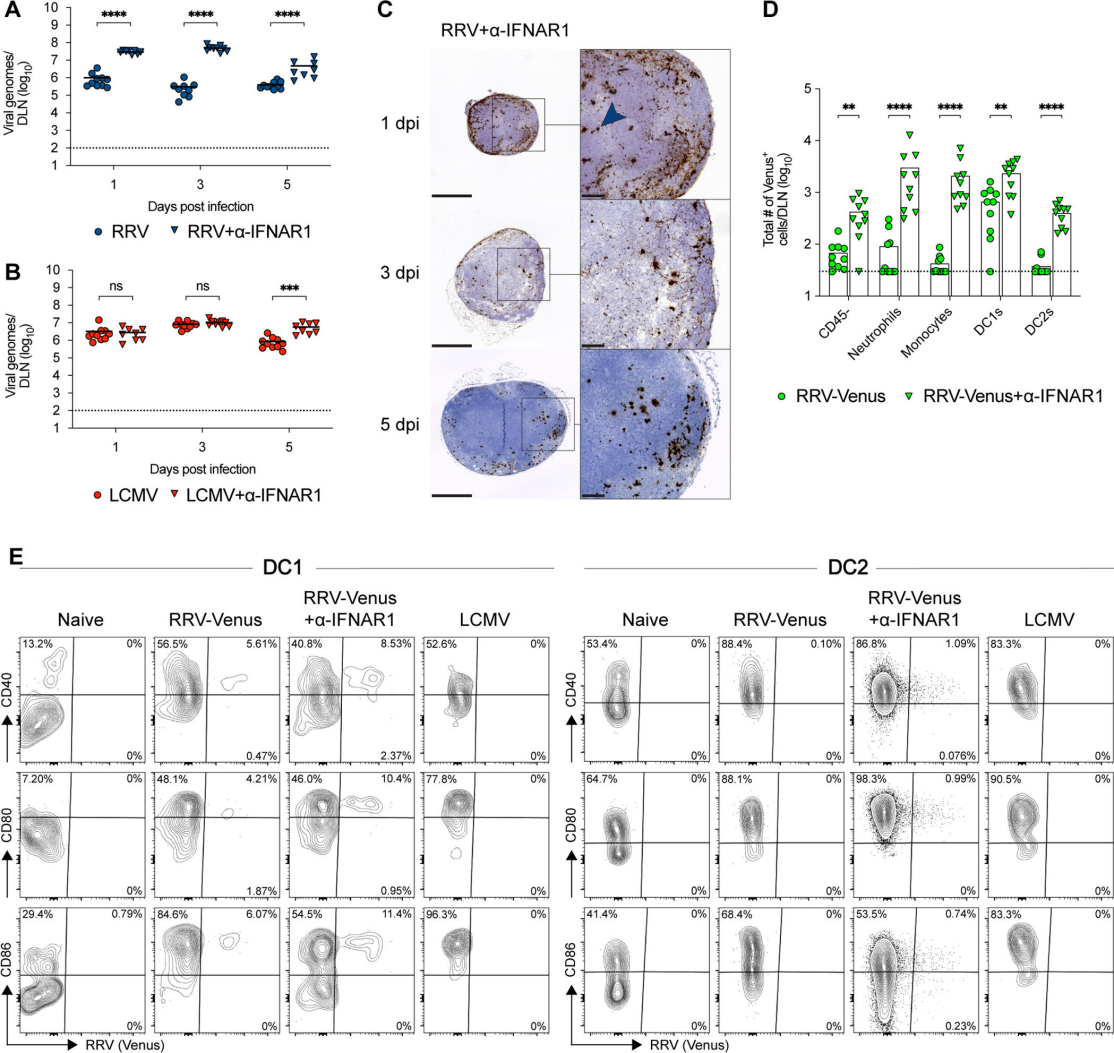
RRV infection in the DLN after local treatment of the anti-IFNAR1 blocking antibody. Three- to 4-week-old male WT C57BL/6 J mice were inoculated in the left footpad with 10^3^ FFU of RRV or LCMV, with or without anti-IFNAR1 mAb treatment (α-IFNAR1). DLNs were harvested at 1, 3, or 5 dpi. (**A**) RRV or (**B**) LCMV RNA in the DLN titered by qRT-PCR (*n* = 8–10 mice per group, three experiments). (**C**) Representative images of RRV RNA localization after anti-IFNAR1 mAb treatment in the DLN at 1, 3, or 5 dpi, as stained by *in situ* hybridization (scale bars: 500 µm) and high-magnification insets (scale bars: 100 µm) (*n* = 3 mice per group, three experiments). Blue arrow, RRV RNA. (**D–F**) Three- to 4-week-old male WT C57BL/6 J mice were inoculated in the left footpad with 4 × 10^5^ FFU of RRV-Venus, with or without anti-IFNAR1 mAb treatment. DLNs were harvested at 36 hours post infection (*n* = 8–10 mice per group, three experiments). (**D**) RRV infection in DLN cells identified by Venus fluorescence using flow cytometry. (**E**) Representative flow cytometry contour plots of DC1 and DC2 Venus fluorescence (RRV infection) and expression of DC activation markers, CD40, CD80, and CD86. Statistical analysis: (**A and B**) Mann–Whitney test comparing between treatment groups at each time point; (**D**) Mann–Whitney test with Holm–Sidak’s post-test comparing between treatment groups; ns = not significant, **P < 0.01, ***P < 0.001, and ****P < 0.0001. Bars and column heights indicate mean values; dotted lines indicate the LOD.

To begin to define why anti-IFNAR1 treatment augments CD8^+^ T-cell priming, we measured viral RNA levels in the DLN. Blockade of type I IFN signaling resulted in 10 to 100-fold higher levels of RRV RNA (*P* < 0.0001) in the DLN between 1 and 5 dpi compared to treatment with an isotype control antibody (Fig. 6A). However, anti-IFNAR1 treatment did not affect LCMV RNA levels in the DLN at 1 and 3 dpi, although at 5 dpi, levels were sixfold higher (*P* < 0.001) compared to isotype control mAb treatment (Fig. 6B); this relatively small effect seen with LCMV may be because it efficiently evades type I IFN responses in mice (28, 29). We next determined the spatial location of RRV RNA after anti-IFNAR1 treatment using *in situ* hybridization. In animals treated with anti-IFNAR1, the RRV RNA signal was readily apparent through day 5 (Fig. 6C), and infected cells were present throughout the DLN, including in the paracortical regions where CD8^+^ T cells localize.

Since RRV appeared to infect additional regions of the DLN in the context of anti-IFNAR1 treatment, we assessed its tropism for antigen-presenting cells using a genetically modified virus that expresses the Venus fluorescent protein (RRV-Venus). DLNs were harvested at 2 dpi and characterized for infection (i.e., Venus expression) by flow cytometry. After anti-IFNAR1 treatment, the Venus protein signal increased in several myeloid cells in the DLN (Fig. 6D; Fig. S5A), including both DC1s (CD11c^+^ MHC-II^+^ Xcr1^+^) and DC2s (CD11c^+^ MHC-II^+^ Sirpα^+^), although we cannot definitively distinguish direct infection from phagocytosis of infected cells using this assay. To determine whether the anti-IFNAR1 blockade affected DC activation, we measured the levels of activation markers CD40, CD80, and CD86 after RRV infection by flow cytometry. Irrespective of anti-IFNAR1 mAb treatment, Venus^+^ DC1s and DC2s showed increased expression of activation markers compared to DCs from naïve mice, with levels that were similar to those of DCs activated in the context of LCMV infection (Fig. 6E).

### Increased CD8^+^ T-cell priming with anti-IFNAR1 mAb treatment is primarily dependent on DCs

**Fig 7:**
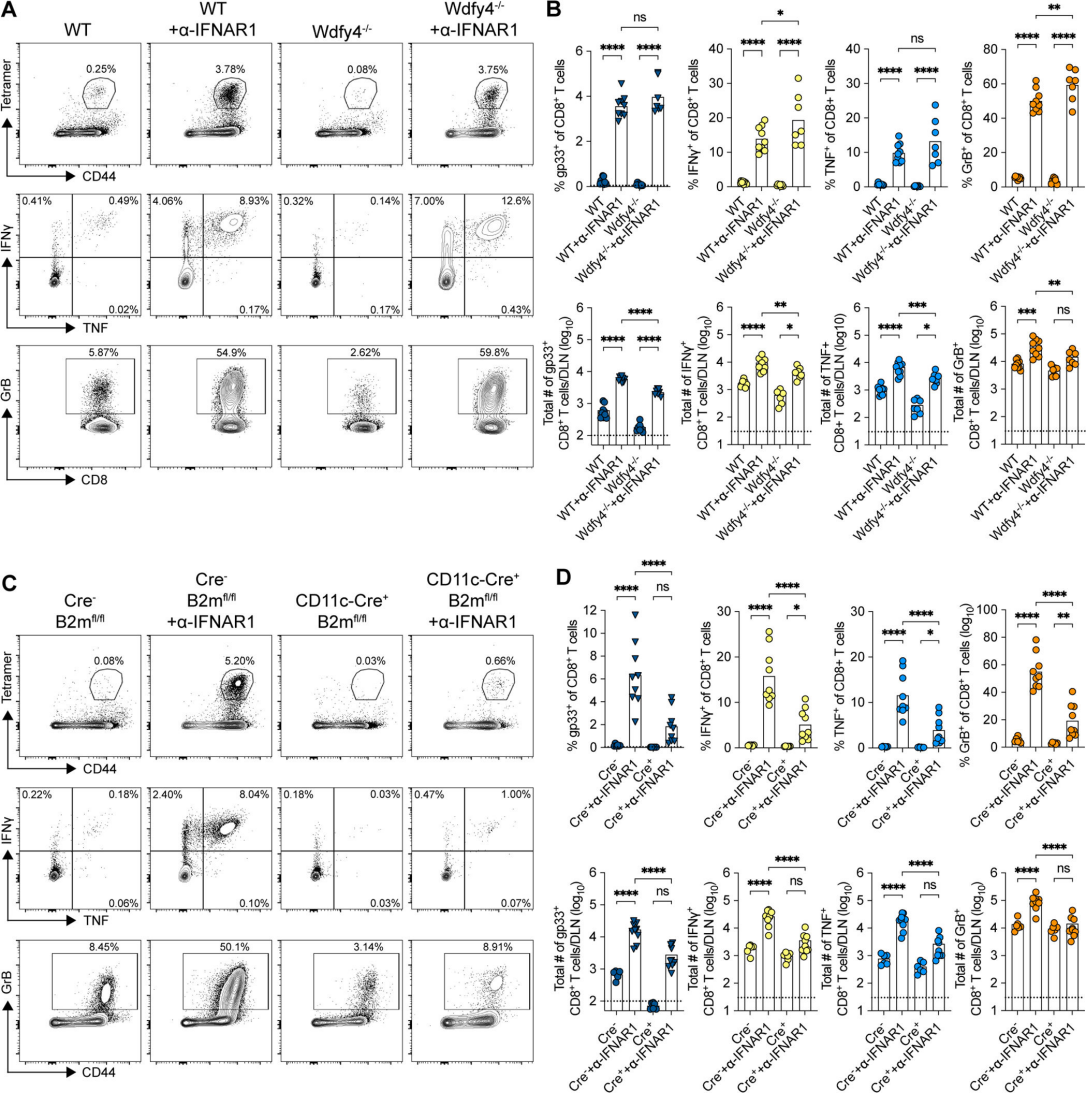
Increased CD8^+^ T-cell priming with anti-IFNAR1 mAb treatment depends on CD11c^+^ DCs. Three-to 4-week-old male and female WT C57BL/6 J or indicated KO mice were inoculated in the left footpad with 10^3^ of RRV-gp33, with or without anti-IFNAR1 mAb (α-IFNAR1) treatment (*n* = 7–10 mice per group, three experiments). At 5 dpi, leukocytes were isolated from the DLN, and CD8^+^ T cells were analyzed by flow cytometry for gp33 tetramer binding, intracellular cytokines after *ex vivo* peptide restimulation, and granzyme B expression. (**A**) Representative flow cytometry contour plots of CD8^+^ T-cell activation in *Wdfy4*^−/−^ mice. (**B**) Summary data of gp33-tetramer^+^, IFNγ^+^, TNF^+^, and GrB^+^ CD8^+^ T cells in *Wdfy4*^−/−^ mice; frequency (top panels) and total numbers (bottom panels). (**C**) Representative flow cytometry contour plots of CD8^+^ T-cell activation in CD11c-Cre *B2m*^fl/fl^ mice. (**D**) Summary data of gp33-tetramer^+^, IFNγ^+^, TNF^+^, and GrB^+^ CD8^+^ T cells in CD11c-Cre *B2m*^fl/fl^ mice; frequency (top panels) and total numbers (bottom panels). Statistical analysis: one-way ANOVA with Holm–Sidak’s post-test; comparisons are within genotypes with and without anti-IFNAR1 mAb treatment and across genotypes with anti-IFNAR1 mAb treatment; *P < 0.05, **P < 0.01, ***P < 0.001, and ****P < 0.0001. Bars and column heights represent mean values; dotted lines indicate the LOD.

Given that anti-IFNAR1 treatment increased RRV infection in the DLN, we hypothesized that the enhanced CD8^+^ T-cell priming might be because DC1s were infected to higher levels and could directly present antigens, or they could more readily cross-present viral antigens to CD8^+^ T cells. To investigate this question, we treated *Wdfy4*^−/−^ mice, which lack a protein essential for DC cross-presentation (30), with anti-IFNAR1 mAb and then infected them with RRV-gp33. Notably, we observed a similar increase in tetramer^+^ (gp33-specific), IFNγ^+^, TNF^+^, and GrB^+^ CD8^+^ T cells in the DLN at 5 dpi of *Wdfy4*^−/−^ and WT mice after anti-IFNAR1 treatment (Fig. 7A and B). Thus, the higher RRV-specific CD8^+^ T-cell response after anti-IFNAR1 treatment was not due to enhanced cross-presentation by DCs.

To address which antigen-presenting cell explains the enhanced CD8^+^ T-cell response when type I IFN signaling is blocked, we infected *Irf8* +32^−/−^ mice (which lack DC1s) (31) or Δ1 + 2 + 3 mice (which lack DC2s) (32). In both sets of mice, we still observed increased RRV-gp33 specific CD8^+^ T-cell responses after anti-IFNAR1 treatment (Fig. S6A and B), suggesting possible functional redundancy. To confirm that DCs were responsible for the enhanced RRV-specific CD8^+^ T-cell response after anti-IFNAR1 treatment, we inoculated CD11c-Cre *B2m*^fl/fl^ mice with RRV-gp33; these mice lack cell surface MHC class I (MHC-I) expression on DCs that express CD11c. Although DC1s and DC2s lost MHC-I expression in these mice, some Ly6C^+^ DC2-like CD11c^+^ MHC-II^+^ Sirpα^+^ cells retain MHC-I expression at 5 dpi, perhaps due to recent differentiation from CD11c^-^ monocytes (Fig. S5B). Importantly, the vast majority of the increased CD8^+^ T-cell responses to RRV-gp33 after anti-IFNAR1 treatment was lost in CD11c-Cre *B2m*^fl/fl^ mice (Fig. 7C and D). These data suggest that both DC1s and DC2s contribute redundantly to the augmented RRV-specific CD8^+^ T-cell response after the type I IFN signaling blockade, and the dependence on MHC-I expression in CD11c^+^ cells suggests that the DCs process antigen directly rather than acquiring peptide–MHC complexes from non-DCs via trogocytosis (33).

### Loss of type I IFN signaling on DC1s can enhance CD8^+^ T-cell polyfunctionality

**Fig 8:**
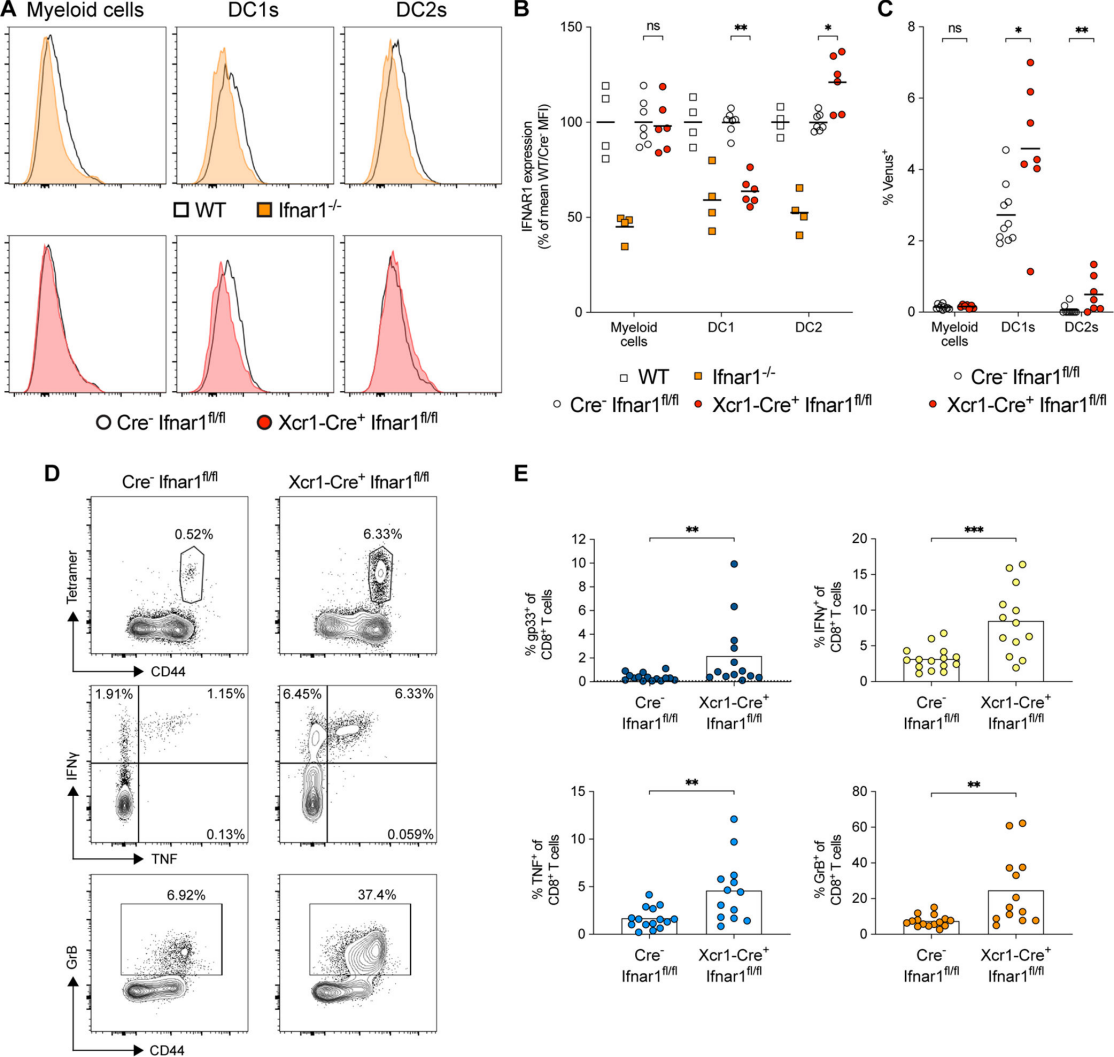
Loss of IFNAR1 signaling in DC1s results in increased CD8^+^ T-cell polyfunctionality after RRV infection. Three- to 4-week-old male and female WT, *Ifnar1*^−/−^, Xcr1-Cre^+^ *Ifnar1*^fl/fl^, or littermate Cre^-^ *Ifnar1*^fl/fl^ mice were inoculated in the left footpad. (**A–C**) At 36 hours post-infection with 4 × 10^5^ FFU of RRV-Venus, leukocytes were isolated from the DLN and analyzed by flow cytometry. (**A**) Representative histograms of IFNAR1 expression on myeloid cells (CD3^-^ NK1.1^-^ B220^-^ CD11b^+^), DC1s, and DC2s. (**B**) Summary data of IFNAR1 median fluorescent intensity (*n* = 4–7 mice per group, two experiments). (**C**) RRV infection of CD11b^+^ myeloid cells, CD11c^+^ Xcr1^+^ DC1s, and CD11c^+^ Sirpα^+^ DC2s identified by Venus fluorescence using flow cytometry (*n* = 7–10 mice per group, two experiments). (**D and E**) At 5 days post-infection with 10^3^ FFU of RRV-gp33, leukocytes were isolated from the DLN, and CD8^+^ T cells were analyzed by flow cytometry (*n* = 13–15 mice per group, four experiments). (**D**) Representative flow cytometry contour plots of CD8^+^ T-cell activation in Xcr1-Cre *Ifnar1*^fl/fl^ mice. (**E**) Summary data of gp33-tetramer^+^, IFNγ^+^, TNF^+^, and GrB^+^ CD8^+^ T cells in Xcr1-Cre *Ifnar1*^fl/fl^ mice. Statistical analysis: (**B and C**) Mann–Whitney test with Holm–Sidak’s post-test comparing between Cre^–^ and Cre^+^ mice for each cell type; (**E**) Mann–Whitney test; ns = not significant, *P < 0.05, **P < 0.01, and ***P < 0.001. Bars and column heights represent mean values; dotted lines indicate the LOD.

Given that LCMV infects DCs (25) and induces a robust antiviral CD8^+^ T-cell response, we hypothesized that direct infection of DCs by RRV might promote maturation of effector CD8^+^ T-cell responses more efficiently. To test this idea, we inoculated Xcr1-Cre^+^ *Ifnar1*^fl/fl^ mice with RRV-gp33; DC1s from these mice specifically lacked IFNAR1 expression (Fig. 8A and B) and were more susceptible to RRV infection than Cre– littermate controls (Fig. 8C). At 5 dpi, RRV-infected Xcr1-Cre^+^ *Ifnar1*^fl/fl^ mice had larger fractions of polyfunctional, gp33-specific CD8^+^ T cells in the DLN than Cre– mice (Fig. 8D and E). Collectively, these results indicate that the antiviral effects of type I IFN signaling suppress RRV infection in DC1s and limit CD8^+^ T-cell priming.

## DISCUSSION

Persistence of alphavirus infection, RNA, and/or antigen contributes to the chronic joint and musculoskeletal tissue inflammation that can last for months. In contrast to many other acute RNA virus infections, in mice, CD8^+^ T cells do not appear to control RRV infection in lymphoid or joint-associated tissues. The differences between the CD8^+^ T-cell response to RNA viruses that do or do not rely on these cells for clearance, however, have not been fully explored. Using RRV and LCMV strains with shared immunodominant gp33 epitopes, we observed differences in the kinetics and magnitude of the CD8^+^ T-cell response in the DLN. Single-cell RNA sequencing showed that fewer antigen-specific CD8^+^ T cells after RRV infection become activated to the same effector state, as seen in most antigen-specific cells after LCMV infection. Consistent with this observation, and in contrast to LCMV, we observed a lower level of RRV RNA in the DLN, including a lack of infection in T-cell zones. To increase the RRV antigen burden in the DLN, we treated mice locally with a blocking anti-IFNAR1 antibody to decrease the expression of antiviral genes. With greater infection in the foot and DLN, the CD8^+^ T-cell response against RRV was improved with numbers similar to those of LCMV. Although both DCs and other myeloid cells showed higher levels of infection after anti-IFNAR1 mAb treatment, experiments with knockout and conditional knockout mice showed that (i) CD11c^+^ DCs were principally responsible for the enhanced RRV-specific CD8^+^ T-cell response; (ii) DC cross-presentation did not explain this enhanced response; (iii) DCs appeared to be directly infected; and (iv) loss of type I IFN signaling in DC1s was sufficient to augment the polyfunctionality of the RRV-specific CD8^+^ T-cell response. Together, these data suggest that the CD8^+^ T-cell response against RRV is limited by type I IFN signaling, which restricts RRV infection in the DLN, viral antigen accumulation in the paracortical regions, and efficient CD8^+^ T-cell priming.

Although the initial course of RRV infection is like many other acute arbovirus infections, viral persistence suggests a defect in the adaptive immune response. Antibodies have key roles in controlling infection by RRV and other arthritogenic alphaviruses, as mice lacking mature B cells develop sustained viremia and foot swelling (14, 34). Mice generate high-affinity neutralizing antibodies against alphaviruses that also have protective Fc effector functions (35, 36), and exogenous treatment with mAbs protects even highly susceptible *Ifnar1*^−/−^ mice (37). While antibodies are essential for combating alphaviruses, they do not prevent persistence (38). This may be because arthritogenic alphaviruses can transmit through intercellular extensions that evade antibody neutralization (39, 40), or because defective, replicating alphavirus RNAs are generated, which lose their ability to express structural proteins on the cell surface that can be recognized by inhibitory antibodies (41). Cell-to-cell spread or defective viral genome replication instead might be contained by cytolytic CD8^+^ T-cell responses.

It has not been clear why CD8^+^ T cells are ineffective in controlling infection by RRV, CHIKV, and possibly other alphaviruses, although induction of immunosuppressive myeloid cells that inhibit antiviral T cells has been reported (42). In our study, RRV-specific CD8^+^ T cells are detectable by day 5 in the DLN, and restimulation with peptide *ex vivo* shows that polyfunctional CD8^+^ T cells can be generated. Moreover, CD8^+^ T cells from the DLN of RRV-infected animals can lyse peptide pulsed cells *ex vivo*, and CHIKV-specific CD8^+^ T cells can clear peptide-pulsed cells *in vivo* (18). Notwithstanding this activity, in the DLN, at 5 dpi, we observed a more than tenfold lower response of gp33-specific CD8^+^ T cells after subcutaneous RRV-gp33 compared to LCMV inoculation despite similar levels of infection in the foot. While some of this phenotype could be due to active disruption of MHC-I surface display and antigen presentation, as recently described for the nsP2 protein of CHIKV (43), our single-cell RNA sequencing of adoptively transferred P14 transgenic CD8^+^ T cells showed a distinct transcriptional trajectory after RRV infection that does not reach terminally differentiated effector state as quickly as after LCMV infection.

Based on the analysis of viral RNA levels and location in the DLN, we speculated that the differences in P14 transgenic CD8^+^ T-cell activation after RRV-gp33 and LCMV infection might be due to disparities in antigen captured or produced by DCs in the paracortical T-cell zones. Whereas LCMV can use α-dystroglycan that is expressed on DCs as a receptor to facilitate entry and infection (25, 44), MXRA8, a primary receptor for RRV, CHIKV, and other arthritogenic alphaviruses, is not expressed on DCs (45), although other receptors may exist (46). Beyond entry pathways, cellular tropism of viruses can also be controlled by type I IFN signaling, especially in immune cells (47–50). Indeed, at baseline, few myeloid cells were infected with RRV in the DLN. To create an environment that more closely mimicked LCMV infection, with greater RRV infection and antigen in the DLN, we locally administered a blocking anti-IFNAR1 antibody at the time of infection. While having little effect on LCMV infection or the LCMV-specific CD8^+^ T-cell response, this treatment resulted in enhanced RRV infection in myeloid cells in the DLN and increased the polyfunctional RRV-specific CD8^+^ T-cell response with kinetics approaching that of LCMV.

Blockade of type I IFN signaling increased RRV CD8^+^ T-cell responses and did not have a detrimental effect on LCMV-induced responses. These results might be considered unexpected, given that CD8^+^ T-cell priming is thought to require T-cell receptor cross-linking, co-stimulation by DC receptors, and a third signal, which can be through type I IFN signaling (51). Indeed, in prior studies, *Ifnar1*^−/−^ CD8^+^ T cells expanded less than WT CD8^+^ T cells in response to LCMV infection (52). It is possible that we did not observe this impairment with anti-IFNAR1 mAb treatment because the signaling blockade was incomplete, relative to a genetic knockout. Alternatively, anti-IFNAR1 mAb treatment may help correct a functionally overactive innate immune response, such as that occurring during the chronic stage of LCMV clone 13 infection, where the IFNAR1 blockade improved CD8^+^ T-cell viral control (53, 54). Besides their direct action on CD8^+^ T cells, type I IFNs can also stimulate DCs (55). A conditional deficiency of IFNAR1 expression in DCs resulted in poor antitumor CD8^+^ T-cell responses that prevented rejection (56), although much of this response was mediated by DC1 cross-presentation and antiviral IFN-stimulated genes are less relevant (30, 57). For conditions where antigen load or replication is affected by type I IFN, such as with a replicating viral vector, the blockade appeared to enhance the vaccine response (58). In our study, we observed greater RRV-specific CD8^+^ T-cell responses with anti-IFNAR1 mAb treatment, and this was associated with an increase in RRV^+^ DCs.

As local anti-IFNAR1 treatment did not qualitatively affect the expression of DC activation markers, we speculated that the increased CD8^+^ T-cell response after RRV infection was due to more antigen presentation by DCs in the DLN. Although RRV infection was increased in multiple cell types after anti-IFNAR1 mAb treatment, we observed a similarly augmented CD8^+^ T-cell response in WT and *Wdfy4*^−/−^ mice, the latter of which lack DC cross-presentation (30). Consistent with these data, a 1,000-fold higher inoculating dose of RRV, by itself, was insufficient to improve the antigen-specific CD8^+^ T-cell response. The presence of DC1 or DC2 was not specifically required for the enhanced phenotype in anti-IFNAR1 mAb-treated mice, whereas the loss of MHC-I expression on CD11c^+^ DCs markedly reduced the CD8^+^ T-cell response. Indeed, direct infection of DCs appeared to be a major contributor to priming as specific deletion of IFNAR1 expression from DC1s was sufficient to augment the polyfunctionality of the RRV-specific CD8^+^ T-cell response.

### Limitations of the study

We acknowledge several limitations in this study. (i) The anti-IFNAR1 antibody could have pleiotropic effects on many cells, which could directly or indirectly impact CD8^+^ T-cell expansion; (ii) defining the direct role of DCs in CD8^+^ T-cell priming is challenging due to the absence of a specific knockout; while CD11c-Cre effectively targets these cells, other cells express CD11c; (iii) the magnitude of the CD8^+^ T-cell response in other tissues was not characterized; (iv) we did not assay clearance of target cells *in vivo*; and (v) studies on the effect of increased CD8^+^ T-cell priming on alphavirus RNA persistence after type I IFN blockade remain to be performed, although they may be complicated by the increased tissue viral burden level when type I IFN signaling is blocked or absent as well as additional CD8^+^ T cell evasion mechanisms at the infected target cell level including nsP2-dependent downregulation of MHC-I (43).

Overall, we demonstrate that an efficient CD8^+^ T-cell response during RRV infection is limited in part by the amount of antigen in the DLN and level of infection of antigen-presenting DCs. Type I IFN induction of antiviral ISGs inhibits tropism for these cells, but when this inhibition is blocked, infected DCs can drive the expansion and maturation of CD8^+^ T cells. This strategy of avoiding replication in antigen-presenting cells allows RRV to delay CD8^+^ T-cell responses. While much remains to be unraveled about the basis of alphavirus persistence in musculoskeletal tissues, strategies to modulate CD8^+^ T-cell effector responses in the acute phase might limit the transition of this infection from the acute to chronic phase.

## MATERIALS AND METHODS

### Cells

BHK-21 (ATCC CCL-10) and Vero (ATCC CCL-81) cells were cultured at 37°C in Dulbecco’s modified Eagle medium (DMEM; Gibco #11995-040), supplemented with 10% fetal bovine serum, 100 U/mL of penicillin, 100 µg/mL of streptomycin, and 10 mM HEPES (pH 7.3).

### Viruses

Stocks of RRV (T48 strain) (59), RRV-gp33 (RRV T48 with an in-frame insertion between the capsid and E3 genes of the immunodominant LCMV MHC-I gp33 peptide sequence along with the FMDV 2A protease sequence) (17), RRV-Venus (RRV T48 with an in-frame insertion between the capsid and E3 genes of the Venus fluorescent gene linked to a LEQLE-SIINFEKL-TEW peptide sequence and the FMDV 2A protease sequence) (43), and LCMV (LCMV-Armstrong clone 53b; provided by M. Oldstone at The Scripps Research Institute) were collected from supernatants after a single passage on BHK-21 cells and titered by focus-forming assay.

### Mice

C57BL/6 J WT (#000664), CD8α^−/−^ (#002665), P14 T-cell receptor transgenic (#004694), C57BL/6 CD45.1 (#002014), CD11c-Cre (#008068), *B2m*^flfl^ (#034858), and *Ifnar1*^fl/fl^ (#028256) mice were purchased from the Jackson Laboratory and bred in-house. *Wdfy4*^−/−^ (30), *Irf8* +32^−/−^ (31), Δ1 + 2 + 3 (32), and Xcr1-Cre (60) C57BL/6 mice have been described previously.

### *In vivo* infections

Three- to 4-week-old mice were inoculated with 10^3^ FFU of RRV or LCMV, 10^6^ FFU of RRV if specified, or 5 × 10^4^ FFU of RRV-Venus via the subcutaneous route in the footpad. For experiments with anti-IFNAR1 mAb treatment, mice received 6 mg/kg (~100 µg) of either anti-IFNAR1 mouse-IgG1 (MAR1-5A3; Leinco # I-401) or isotype control mouse-IgG1 mAb specific to the human IFNγ receptor (GIR-208; Leinco #I-443), which was mixed with the virus before footpad injection. At specified time points, mice were anesthetized and euthanized with ketamine and xylazine, and tissues were harvested. For flow cytometric analysis of T cells, DLNs (popliteal) were pressed through a 70-µm cell strainer to obtain single-cell suspensions. A portion of cells were plated separately for intracellular cytokine analysis after a 4-h stimulation at 37°C with 1 µg/mL of gp33 (KAVYNFATM), OVA (SIINFEKL), or RRV peptides (HSHSNVATL, VGFPYKAHI, AGIINASNI, or VGPNFSTV) in RPMI 1640 (Thermo Fisher #11875093) media supplemented with 10% FBS and 5 µg/mL brefeldin A (Biolegend). For flow cytometric analysis of myeloid cells, DLNs were initially minced and digested for 30 minutes in DMEM supplemented with 125 µg/mL of Liberase TL (Roche) and 50 µg/mL of DNase. For viral titer analysis, mice were perfused via intracardial injection with 20 mL of sterile PBS before tissues were dissected and stored in 2-mL tubes at −80°C.

### Viral RNA quantification

Frozen tissues were weighed and homogenized with ceramic beads in 250 to 1.000 µL of DMEM supplemented with 5% FBS using a MagNA Lyser Instrument (Roche). Viral RNA was quantified after extraction from 50 µL of the clarified homogenate using an MagMax viral RNA isolation kit (Thermo Fisher) and Kingfisher Flex (Thermo Fisher) using a one-step RT-qPCR kit on a QuantStudio 6 real-time PCR system (Thermo Fisher). Viral genome copies were quantified using a standard curve of RNA isolated from viral stocks and published primers and probes specific to RRV nsP3 (61) or LCMV GP (62).

### Flow cytometry

Single-cell suspensions were blocked for nonspecific FcγR binding (1:200 dilution; clone 93; Biolegend). T cells were stained with anti-CD8α (1:200 dilution; clone 52-6.7; Biolegend) and gp33-loaded H2-D^b^ (KAVYNFATM; Immunomonitoring Laboratory, Washington University) that was tetramerized with PE-conjugated streptavidin (Biolegend) for 15 minutes at 37°C. Subsequently, at 4°C, cells were incubated with the fixable viability dye (1:1,000 dilution; e506; eBiosciences) and antibodies to CD45 (1:200 dilution; clone 30-F11; Biolegend), CD3 (1:100 dilution; clone 145-2C11; Biolegend), CD4 (1:200 dilution; clone RM4-5; Biolegend), and CD44 (1:200 dilution; clone IM7; Biolegend). Other cells were stained with antibodies against B220 (1:200 dilution; clone RA3-6B2; Biolegend), CD69 (1:200 dilution; clone H1.2F3; Biolegend), CD11b (1:400 dilution; clone M1/70; Biolegend), Ly6C (1:400 dilution; clone HK1.4; Biolegend), Ly6G (1:200 dilution; clone 1A8; Biolegend), NK1.1 (1:200 dilution; clone PK136; Biolegend), CD11c (1:200 dilution; clone N418; Biolegend), MHC-I (1:200 dilution; clone AF6-88.5; Biolegend), and MHC-II (1:400 dilution; clone M5/114.15.2; Biolegend). IFNAR1 was stained with biotinylated (Thermo Scientific #A39256) anti-IFNAR1 mouse-IgG1 (MAR1-5A3; Leinco # I-40), followed by streptavidin–APC (Biolegend). Cells were washed and fixed with 4% PFA for 10 minutes at room temperature. T cells were subjected to fixation and permeabilization using a kit (BD Biosciences #554714) and stained at 4°C with antibodies to IFNγ (1:200 dilution; clone XMG1.2; eBiosciences), TNF-α (1:200 dilution; clone MP6-XT22; eBiosciences), and GrB (1:200 dilution; clone QA16A02; Biolegend). Cells were washed and resuspended in PBS, supplemented with 2% FBS, and Precision Count Beads (Biolegend) were added. Samples were processed on a Cytek Aurora flow cytometer and analyzed using Flowjo v10 (BD Biosciences).

### *Ex vivo* T-cell cytotoxicity assay

Effector CD8^+^ T cells were negatively selected from DLNs at 5 dpi using an EasySep Mouse CD8^+^ T-Cell Isolation Kit (STEMCELL technologies #19853). We used splenocytes from naive WT mice as target cells; these were obtained after red blood cell lysis with ACK lysing buffer (Gibco #A1049201) and centrifugation. Cells were pulsed with 5 µg/mL of the gp33 peptide (KAVYNFATM) or OVA peptide (SIINFEKL) for 30 minutes and labeled with 5 µM or 0.5 µM CellTrace Violet (Thermo Fisher # C34557), respectively, for 15 minutes. Cells were plated at the indicated effector:target ratios in a V-bottom 96-well plate (Corning # 3894), briefly centrifuged at 200 × *g*, and incubated at 37°C for 4 hours before 7-AAD was added to each well. Cell death was assessed by flow cytometry, as described previously (63).

### Single-cell RNA sequencing

CD8^+^ T cells were negatively selected from the spleen of CD45.2 P14 transgenic mice using an EasySep Mouse CD8^+^ T Cell Isolation Kit (STEMCELL technologies #19853). Recipient CD45.1 C57BL/6 mice were adoptively transferred 10^6^ CD45.2 P14 cells via retroorbital injection and then inoculated in the footpad with 10^3^ FFU of RRV-gp33 or LCMV. DLNs (popliteal) were harvested at 5 dpi and pooled (*n* = 7 mice per group). DLN cells from uninfected P14 mice served as a negative control. For each group, 1.5 × 10^4^ total DLN cells were combined with an enriched population of 5 × 10^3^ P14 CD8^+^ T cells isolated using an EasySep Mouse CD8^+^ T Cell Isolation Kit (STEMCELL technologies #19853), followed by cell sorting for CD45.2^+^ P14 cells using a BD FACSAria II. The combined 2 × 10^4^ cells per group were submitted for single-cell RNA sequencing (Genome Technology Access Center, Washington University). Live cells were sorted into droplets, and libraries were prepared using a Chromium Single-Cell 5' Reagent Kit v2 with TCR enrichment, as described in the manufacturer’s protocol (10 x Genomics) and sequenced on an Illumina next-generation sequencing machine. Raw fastq files were processed with Cellranger 7.0.1 and imported into R (v4.3.1) and analyzed using the Seurat R package (v4.4.0) Quality control measures excluded genes that were expressed in less than three cells, cells with less than 200 genes detected, and cells with greater than 5% mitochondrial gene content. Cells predicted to be doublets by the DoubletFinder R package (v2.0.3) (64) were also excluded from analysis. P14 CD8^+^ T cells were identified by 5' TCR sequencing with 99% identity to the variable regions of the published TCRα and the TCRβ P14 sequence (65). The samples were integrated and then normalized using Seurat’s SCTransform function while regressing percent mitochondrial gene content and cell cycle scoring. Thirty principal components were calculated, clusters were determined via the Louvian algorithm, and dimensional reduction was performed and visualized with a uniform manifold approximation and projection (UMAP). Z-scores were calculated by subtracting the average expression in the naive cluster 0 from each sample’s normalized RNA expression values, followed by division by the standard deviation of expression values within each gene across all samples.

### Histology and viral RNA *in situ* hybridization

Dissected DLNs and feet were fixed in 15 x volume of 10% neutral buffered formalin for 24 hours at room temperature. Feet were decalcified with daily changes of 14% EDTA (free acid) for 14 days. Tissues were then washed with water, dehydrated in 30% and 50% ethanol washes, and stored in 70% ethanol at 4°C before paraffin embedding and sectioning (Musculoskeletal Histology and Morphometry Core, Washington University). Viral RNA was visualized using an RNAscope 2.5 Brown *in situ* hybridization kit (ACD). Tissue sections were deparaffinized and prepared as described in the manufacturer’s protocol (ACD document #322310). Samples were treated with virus specific probe V-RossRiver-T48-E2E3 (ACD #494871) targeting RRV subgenomic RNA or V-LCMV-S (ACD #493781) targeting the LCMV S genome segment. After detection, slides were counterstained with hematoxylin and imaged on a Hamamatsu NanoZoomer 2.0-HT slide scanner.

### CD8^+^ T-cell epitope prediction

To identify peptide candidates for MHC-I restricted CD8^+^ T-cell recognition in C57BL/6J (H2^b^ haplotype) mice, we scanned the amino acid sequences of the RRV structural polyprotein (GenBank Accession ACV67002.1) and nonstructural polyprotein (GenBank Accession ACV67001.1) for peptides predicted to bind H2-K^b^ and H2-D^b^. We used the NetMHCPan 4.1 EL method (66) as implemented and recommended in the IEDB Analysis Resource (67) and considered all peptides of lengths 8–13. The 19 soluble peptides that had normalized percentile rank scores within the top 0.06 percentile were synthesized and tested as high-likelihood candidates for MHC-I binding.

### Statistical analysis

Statistical significance was assigned when *P* values were < 0.05 using Prism version 10.2.3 (GraphPad). Biological experiments, comparison groups, statistical tests, and number of animals (*n*) are indicated in the figures and figure legends.

## Data Availability

All data supporting the findings of this study are available within the paper and from the corresponding author upon request. This paper does not include the original code. The single-cell RNAseq data have been deposited in NCBI’s Gene Expression Omnibus and are accessible through GEO Series accession number GSE275970. Any additional information required to reanalyze the data reported in this paper is available from the corresponding author upon request.

## SUPPLEMENTAL MATERIAL

Supplemental Figures mbio.02930-24-s0001.pdf Fig. S1 to S6.

ASM does not own the copyrights to Supplemental Material that may be linked to, or accessed through, an article. The authors have granted ASM a non-exclusive, world-wide license to publish the Supplemental Material files. Please contact the corresponding author directly for reuse.
