# Supplementary material for: Type I interferon signaling in dendritic cells limits direct antigen presentation and CD8+ T cell responses against an arthritogenic alphavirus
Source: mBio. 2024 Nov 13;15(12):e02930-24. doi: 10.1128/mbio.02930-24 (PMC11633147; doi:10.1128/mbio.02930-24)
Supplement: Supplemental Figures — Fig. S1 to S6. [file mbio.02930-24-s0001.pdf]

SUPPLEMENTAL FIGURES AND LEGENDS

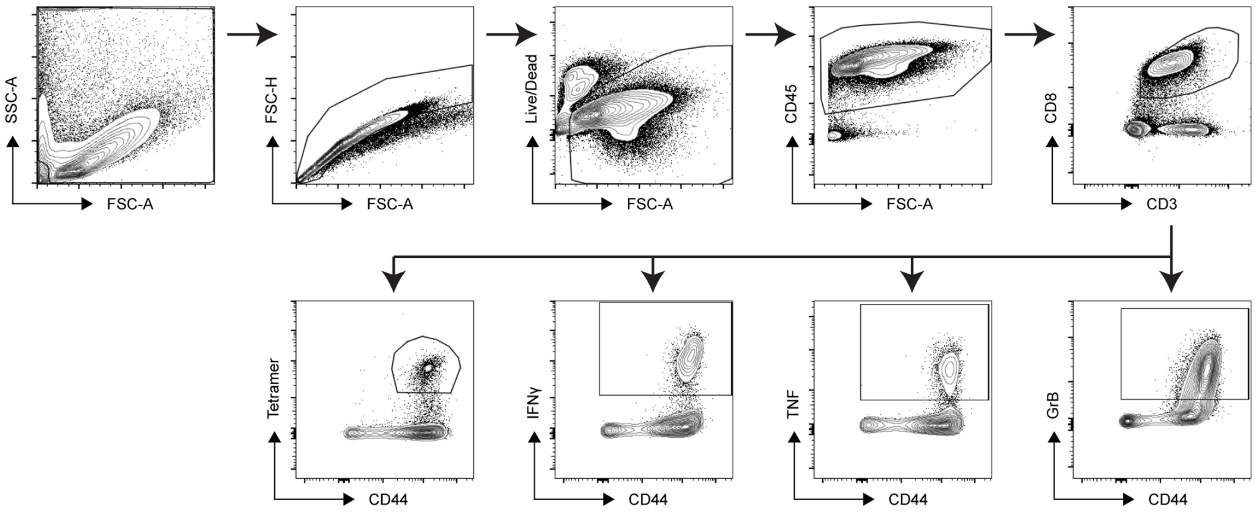

Figure S1. Gating strategy of CD8⁺ T cells in the DLN. DLN cells were harvested at 5 dpi, stained with antibodies against the indicated antigens, and analyzed by flow cytometry. One representative experiment of 3 is shown. **Related to Figures 2, 5, 7, and 8.**

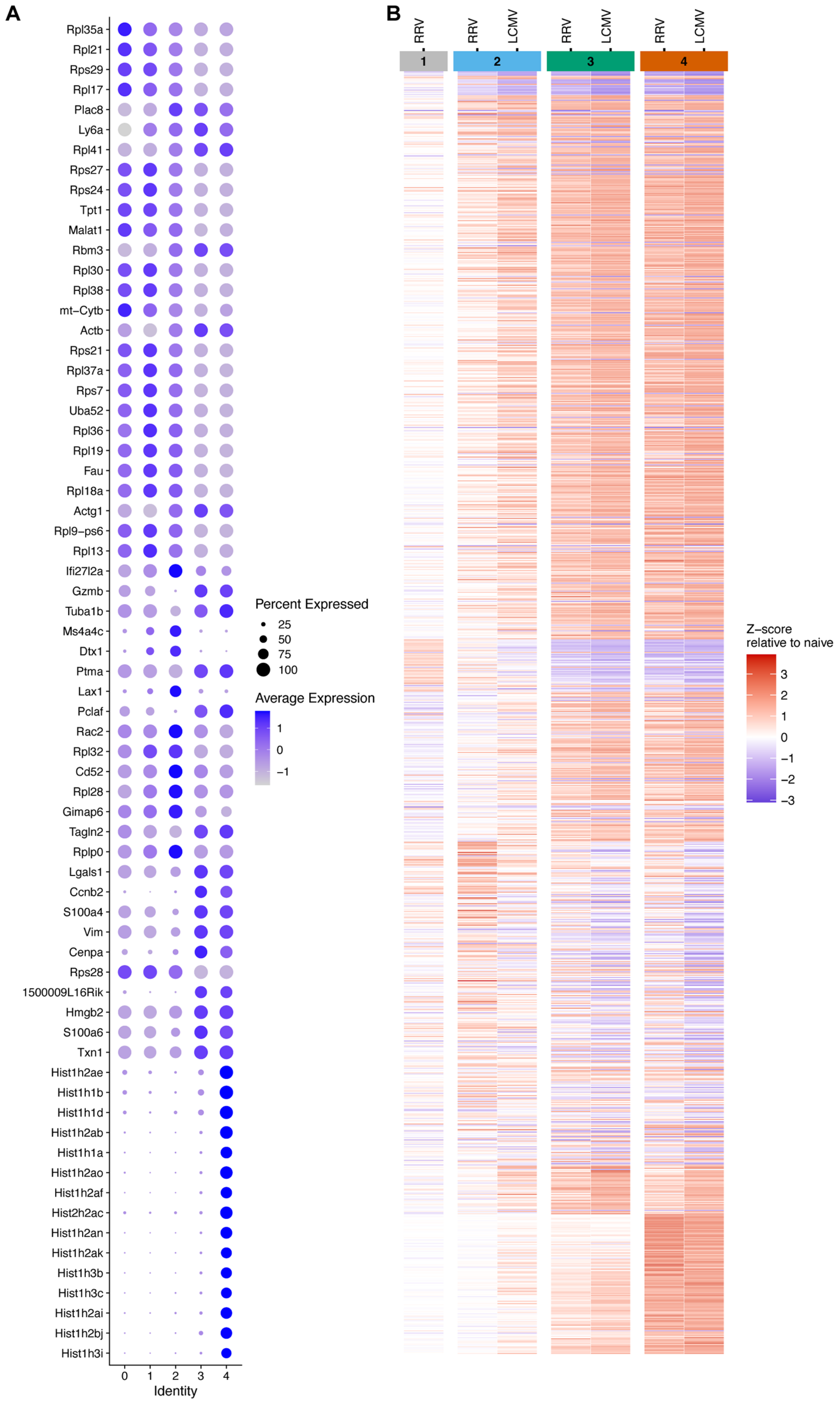

Figure S2. Expression of cluster-determining differentially expressed genes. (**A**) Expression of the top 15 gene determinants of each cluster, per cluster. (**B**) Change in expression of the top

500 gene determinants of each cluster, per cluster/sample, relative to mean expression in cluster 0 of the naive sample. **Related to Figure 3**.

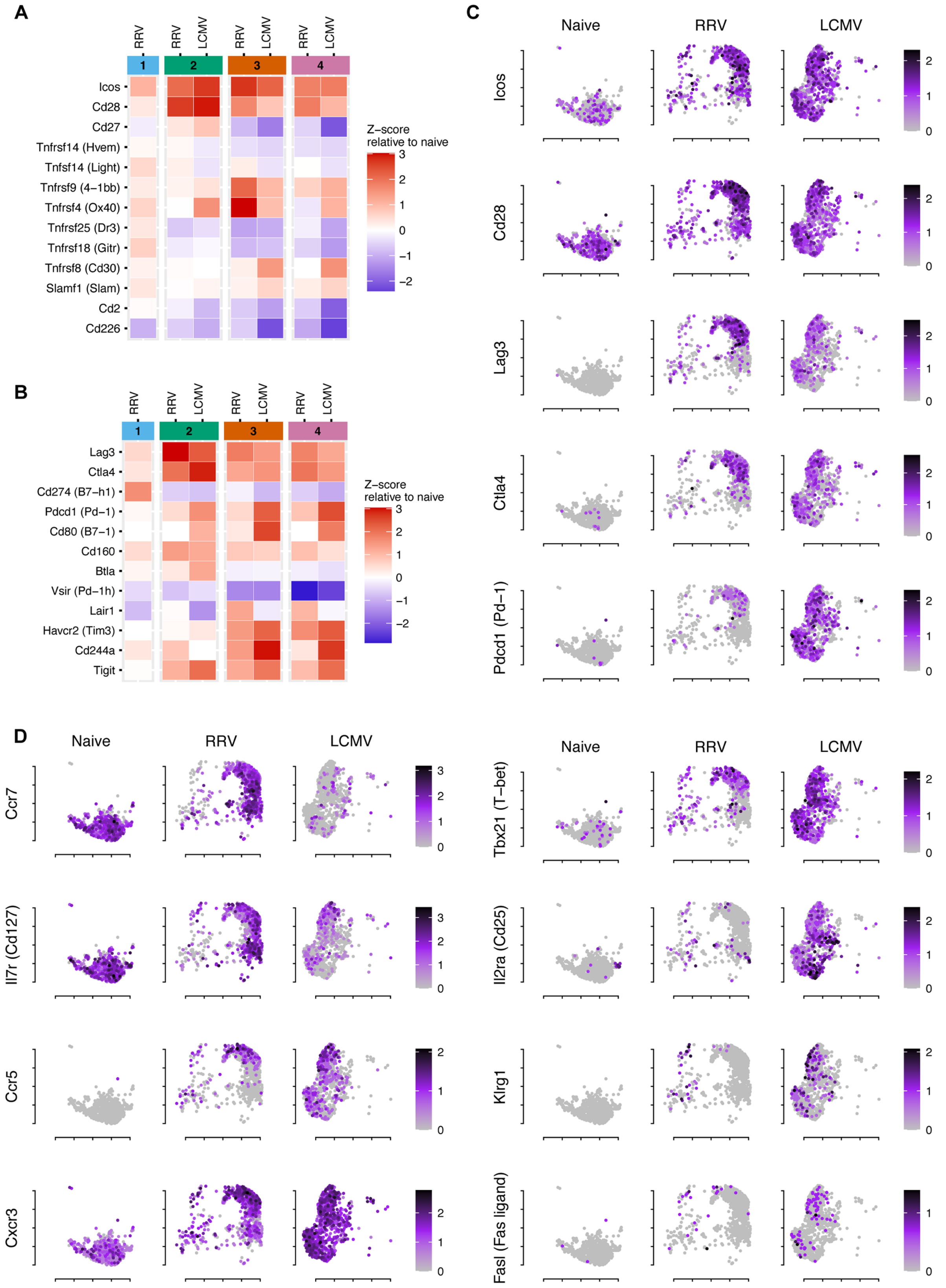

Figure S3. Expression of costimulatory and inhibitory receptor genes and other selected genes. (**A-B**) Change in expression of co-stimulatory (**A**) and co-inhibitory (**B**) T cell receptor

genes, per cluster/sample, relative to mean expression in cluster 0 of the naive sample. (**C**) Expression of selected co-stimulatory and co-inhibitory receptor genes in $CD8^+$ T cells. (**D**) Expression of additional selected $CD8^+$ T cell activation genes. **Related to Figure 3.**

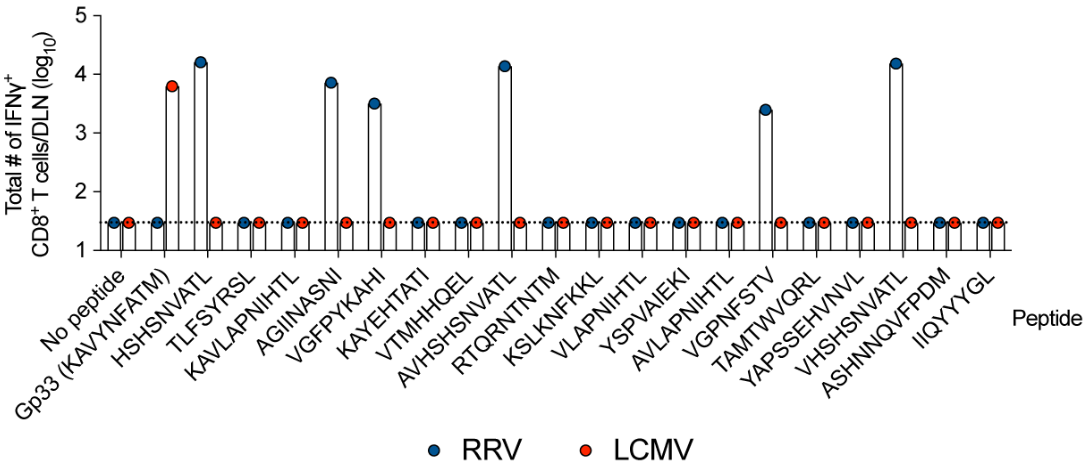

Figure S4. T cell response to predicted RRV-native peptides. DLN cells were harvested at day 5 after RRV or LCMV infection and restimulated *ex vivo* with predicted class I MHC binding RRV peptides for 18 h before intracellular staining for IFNγ and flow cytometry analysis (n = 4 pooled mice per group, 2 experiments). Column height indicates mean values. **Related to Figure 5.**

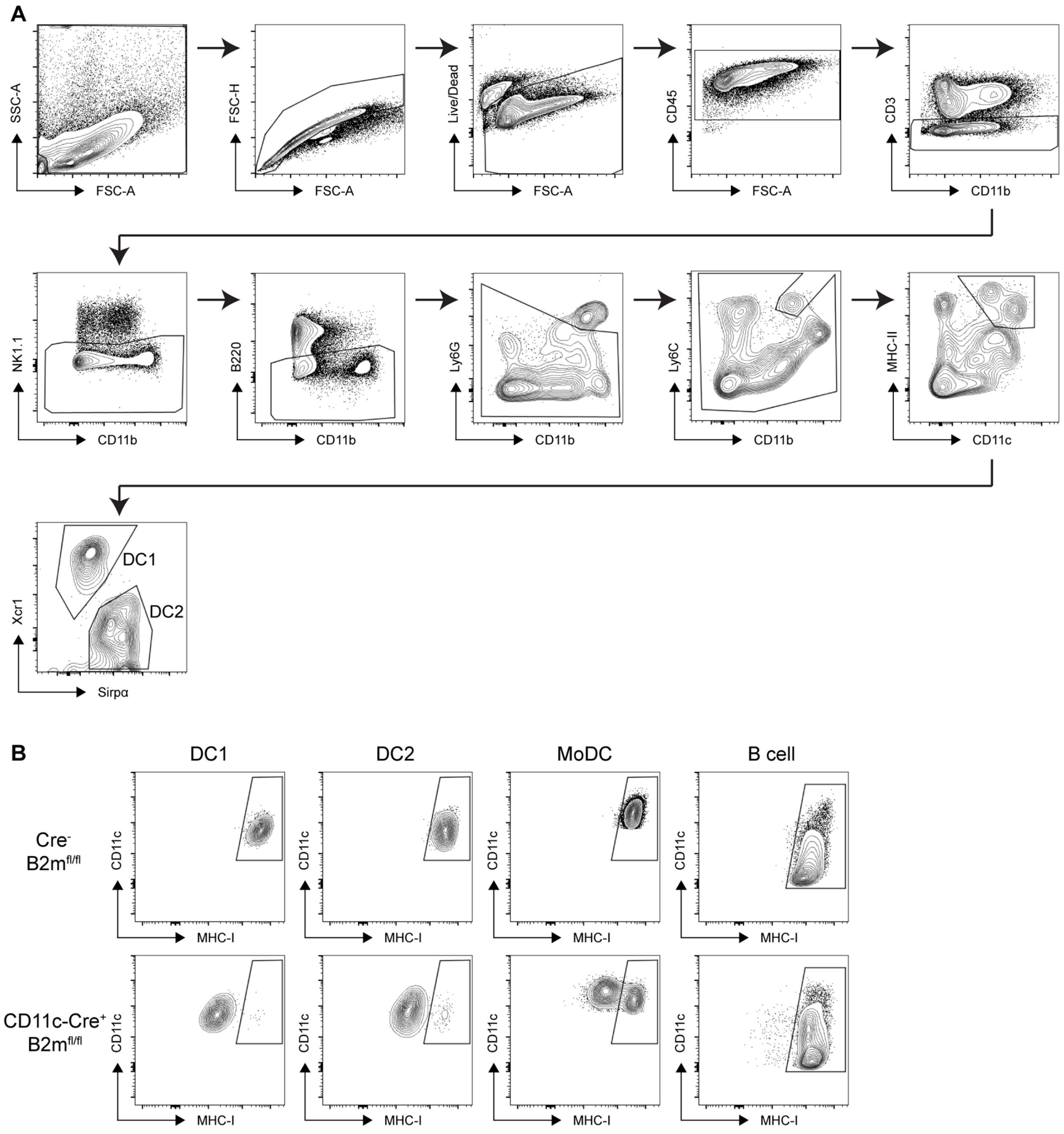

Figure S5. Gating strategy of DC subsets in the DLN and β2-microglobulin expression on DCs in CD11c-Cre^{+} *B2m*$^{fl/fl}$ mice. (**A**) Gating strategy. DLN cells were harvested at 36 h post infection, stained with antibodies against the indicated antigens, and analyzed by flow cytometry. One representative experiment of 3 is shown. (**B**) β2-microglobulin expression in DC1, DC2, monocyte-derived DCs (Ly6C^{+} CD11c^{+} MHC-II^{+} Sirpα^{+}), and B220^{+} B cell populations in the DLN from indicated Cre^{-} *B2m*$^{fl/fl}$ or CD11c-Cre^{+} *B2m*$^{fl/fl}$ mice as detected by flow cytometry. One representative experiment of 2 is shown. **Related to Figure 6, 7, and 8.**

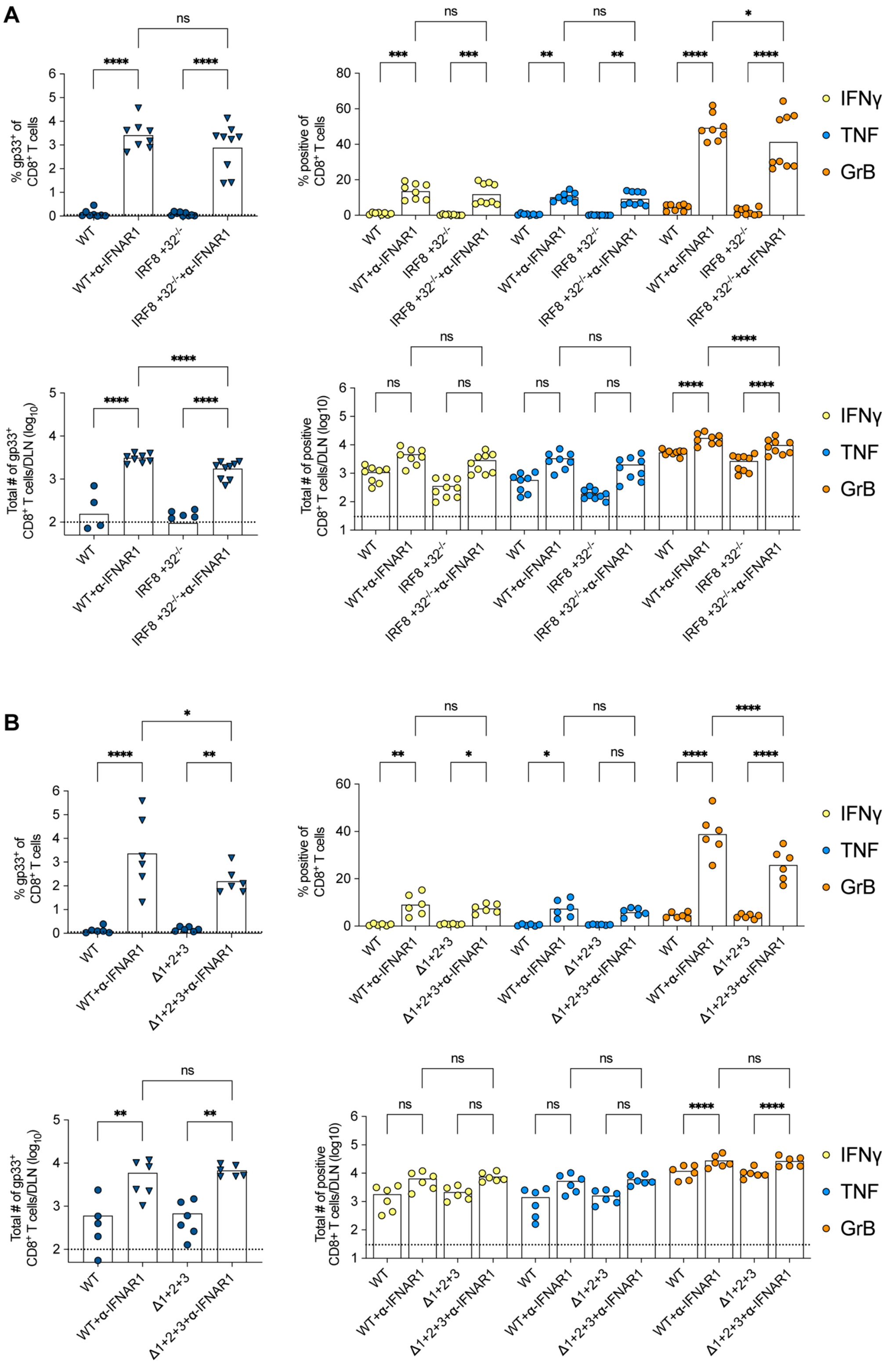

Figure S6. Increased RRV-specific CD8^{+} T cell response is not dependent on individual DC1 or DC2 subsets. Three to 4-week-old male and female WT C57BL/6J or indicated KO mice were inoculated in the left footpad with 10^3 of RRV-gp33, with or without anti-IFNAR1 (α-IFNAR1)

treatment (n = 6-9 mice per group, 3 experiments). At 5 dpi, leukocytes were isolated from the DLN, and $CD8^+$ T cells were analyzed by flow cytometry for gp33 tetramer binding, intracellular cytokines, and granzyme B expression. (**A**) $CD8^+$ T cell functional profiling in *IRF8Δ32* mice that lack DC1s. (**B**) $CD8^+$ T cell functional profiling in Δ1+2+3 mice that lack DC2s. Statistical analysis: one-way ANOVA with Holm-Sidak's post-test; comparisons are within genotypes with and without anti-IFNAR1 and across genotypes with anti-IFNAR1 treatment; * $p < 0.05$, ** $p < 0.01$, *** $p < 0.001$, **** $p < 0.0001$. Bars represent mean values; dotted lines indicate the LOD. **Related to Figure 7.**
